# Genomics analysis of potassium channel genes in songbirds reveals molecular specializations of brain circuits for the maintenance and production of learned vocalizations

**DOI:** 10.1186/1471-2164-14-470

**Published:** 2013-07-11

**Authors:** Peter V Lovell, Julia B Carleton, Claudio V Mello

**Affiliations:** 1 Department of Behavioral Neuroscience, Oregon Health and Sciences University, 3181 Sam Jackson Park Rd L470, Portland, OR, USA

**Keywords:** Learned vocalizations, Songbird, Potassium channel, Genomics, Gene expression, In situ hybridization, Song system

## Abstract

**Background:**

A fundamental question in molecular neurobiology is how genes that determine basic neuronal properties shape the functional organization of brain circuits underlying complex learned behaviors. Given the growing availability of complete vertebrate genomes, comparative genomics represents a promising approach to address this question. Here we used genomics and molecular approaches to study how ion channel genes influence the properties of the brain circuitry that regulates birdsong, a learned vocal behavior with important similarities to human speech acquisition. We focused on potassium (K-)Channels, which are major determinants of neuronal cell excitability.

Starting with the human gene set of K-Channels, we used cross-species mRNA/protein alignments, and syntenic analysis to define the full complement of orthologs, paralogs, allelic variants, as well as novel loci not previously predicted in the genome of zebra finch (*Taeniopygia guttata*). We also compared protein coding domains in chicken and zebra finch orthologs to identify genes under positive selective pressure, and those that contained lineage-specific insertions/deletions in functional domains. Finally, we conducted comprehensive *in situ* hybridizations to determine the extent of brain expression, and identify K-Channel gene enrichments in nuclei of the avian song system.

**Results:**

We identified 107 K-Channel finch genes, including 6 novel genes common to non-mammalian vertebrate lineages. Twenty human genes are absent in songbirds, birds, or sauropsids, or unique to mammals, suggesting K-Channel properties may be lineage-specific. We also identified specific family members with insertions/deletions and/or high dN/dS ratios compared to chicken, a non-vocal learner. *In situ* hybridization revealed that while most K-Channel genes are broadly expressed in the brain, a subset is selectively expressed in song nuclei, representing molecular specializations of the vocal circuitry.

**Conclusions:**

Together, these findings shed new light on genes that may regulate biophysical and excitable properties of the song circuitry, identify potential targets for the manipulation of the song system, and reveal genomic specializations that may relate to the emergence of vocal learning and associated brain areas in birds.

## Background

With the recent availability of a large number of genome sequences for higher vertebrates, there are growing opportunities for understanding how genes have contributed to the evolution and functional organization of brain circuits for complex learned behaviors. Among genes that may have evolved to shape the physiological properties of such circuits, ion channel genes represent highly compelling candidates. Potassium Channel (K-Channel) genes, in particular, are one of the largest and most structurally diverse families of ion channels genes and are known to regulate a wide array of neuronal functions, from resting membrane potential and intrinsic excitability to action potential repolarization and propagation [1-3]. Whereas the fruit fly (*Drosophila melanogaster*) has ~30 K-Channels genes [4,5], in the human genome more than 100 distinct loci have been identified that encode either the structural determinants (i.e. alpha-subunits) or accessory modulatory components (i.e. beta-subunits, channel tetramerization proteins). This vast expansion in vertebrates has been suggested as being related to the evolution of complex organs whose function requires the precise control of membrane excitability, such as the heart and the central nervous system.

Here we describe our use of songbirds to investigate the relationship between the K-Channel gene family and the emergence of a complex learned behavior. Song is a learned vocal behavior that shares many important features with speech and language capabilities in humans [6-9]. The connectivity, physiology and pharmacology of specialized brain areas for vocal learning and production (i.e. song system) have been extensively characterized in male zebra finches, the most tractable model organism for studying the neurobiological basis of vocal learning (see reviews in [10,11]). The recent completion of the zebra finch genome sequence [12], the second avian genome to be sequenced after the chicken [13], has made it possible to search for molecular mechanisms that may have evolved in association with song learning. Furthermore, vocal learning and associated neural circuits are generally lacking in most other vertebrate groups, including the majority of avian orders (e.g. chicken, pigeon, owls, shorebirds, etc.), rodents, and non-human primates. Thus, a comparative genomics approach offers unique opportunities for revealing genomic features and specializations that may relate to the emergence and/or maintenance of vocal learning.

**Figure 1:**
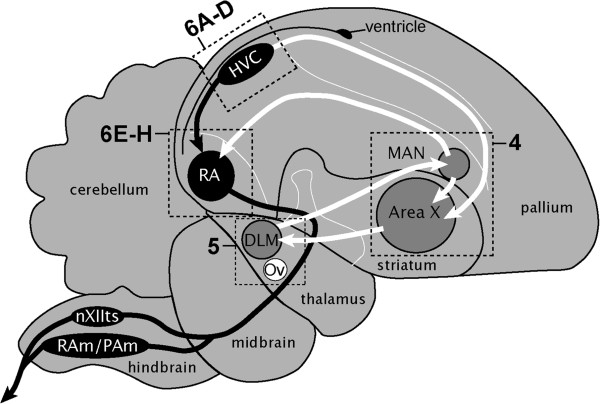
**Major brain areas for vocal learning and singing in zebra finches.** Composite diagram of the songbird brain (parasagittal plane) illustrating the approximate positions and connections of the major nuclei of the song control system. Several related nuclei and connections have been removed for clarity. The song system consists of a direct motor pathway (DMP) for song production (in black) that includes connections from song nucleus HVC to RA, and from RA to brainstem nuclei involved in vocal-motor and respiratory control (nXIIts, RAm, and PAm), and an anterior forebrain pathway (AFP) for song learning (nuclei in grey; projections in white) that includes a cortico-thalmo-cortical loop from Area X in the striatum to thalamic DLM, from DLM to LMAN, and from LMAN back to Area X. Dotted rectangles indicate the approximate positions of the photomicrographs presented in the panels in Figure 4 and Figure 5. See Abbreviations for a complete list of anatomical abbreviations.

In songbirds, vocal learning requires a unique set of forebrain nuclei and projections that are noticeably absent in birds that do not learn to vocalize (Figure 1; [10,11]. This so-called song control system is typically divided into two interconnected pathways: (1) a direct motor pathway (DMP) [14,15], which is essential for the production of learned birdsong (Figure 1, black); and (2) an anterior forebrain pathway (AFP), which forms a cortico-basal ganglia-thalamo-cortical loop, and is essential for song learning [16-18] and adult vocal plasticity [19-22]. This latter pathway is connected to the DMP through projections from HVC to X and LMAN to RA [23-25]. The anatomical separation of these circuits and the discrete nature of their component nuclei greatly facilitate correlating gene expression to specific electrophysiological properties that may underlie various aspects of sensorimotor learning and singing.

Several lines of evidence suggest that K-Channel genes may play important roles in controlling vocal production and learning in songbirds. For example, KCNC2, which encodes a delayed-rectifier type K-Channel that is typically expressed in fast spiking GABAergic interneurons in mammals [26], has been reported as being under strong positive selection in zebra finches as compared to chicken, a non-vocal learning avian species [6,12]. Furthermore, a microarray screening [27] has revealed that specific K-Channel transcripts (e.g. KCNA1, KCNC2, KCND2, KCNF1) are differentially expressed in HVC, a nodal nucleus that provides input into the two main pathways in the song system (Figure 1) and is involved in both the acquisition and production of learned song. Indeed, intracellular recordings can readily discriminate interneurons and projection neurons within HVC based upon differences in membrane resting potential, input resistance, sub-threshold responses, cell excitability, action potential after-hyperpolarization, and synaptic inhibition [28-31]. Nearly all of these properties are likely to be influenced by expression of K-Channel genes.

We reasoned that a comprehensive survey of K-Channel genes in the zebra finch genome and their expression patterns in the brain would provide important insights into the properties of the circuits for production and maintenance of the adult song, indicating molecular targets for the pharmacological and/or genetic manipulation of singing behavior. Starting with the complete complement of K-Channel genes in the human genome, we identified 107 zebra finch loci that encode either structural components of K-Channels (i.e. alpha-, beta-subunits), or channel-specific modifiers. Our analysis revealed some previously unrecognized K-Channel genes that may be unique to non-mammalian vertebrate lineages, as well as others that appear to have been lost in birds or are possibly unique to mammals. We also identified K-Channel genes that possess either songbird-specific protein insertions/deletions compared to chicken and mammals, or that are under high selective pressure (i.e. high dN/dS values). Finally, *in situ* hybridization analysis revealed the broad distribution of K-Channel genes in the zebra finch brain, as well as molecular specializations of key nuclei of the song system, indicating possible roles for K-Channels in production and/or maintenance of adult birdsong. Our study represents the first comprehensive analysis of a family of ion channels in an avian genome and identifies molecular components that likely regulate the biophysical and excitable properties of the song control circuitry. More broadly, our demonstrate that a combined genomics and molecular approach can provide compelling evidence for the involvement of specific gene families in the shaping of brain circuits for complex learned behaviors.

## Results

### Potassium (K-)Channel genes in the zebra finch genome

To identify K-Channel genes in the zebra finch genome, we first searched the HGNC gene and family lists [32], as well as the IUPHAR voltage-gated channel list [33] and defined 123 genes representing all identified K-Channel genes in the human genome. This set included proteins related to the assembly (e.g. alpha subunits) or modulation (e.g. accessory beta subunits) of K-Channels, as well as proteins containing a conserved K-Channel-like tetramerization domain thought to modulate the function of GABA_B_ receptors [34]. We next BLAT-aligned each human gene model to the zebra finch genome using UCSC’s genome browser [35], and used syntenic analysis to define orthologous loci in chicken and other vertebrate organisms. In several cases, the zebra finch gene model required alignment to additional vertebrate genomes (lizard, frog, fish, mouse, human and others) to verify orthology and/or confirm the presence or absence of the gene at different nodes within the vertebrate lineage. We also consistently BLAT-aligned each confirmed ortholog back to the zebra finch genome in order to identify additional loci corresponding to duplicated genes and/or paralogs that were not predicted by Ensembl genebuild (see Methods for details).

Our search resulted in the identification of 107 distinct genes that we believe correspond to the full complement of K-Channel and related genes in the zebra finch genome (Table 1). This includes members of each of the 21 recognized sub-families of K-Channel genes, including representatives of the 6 transmembrane domain (6TM) and calcium-gated channels (e.g. KCNA, HERG, KQT-like, KCNMA), the 2TM channels (e.g. IRK, GIRK), the 4TM channels (e.g. KCNK), the brain cyclic-nucleotide gated channels (e.g. HCN), the K-Channel tetramerization proteins (KCTD), and various accessory subunits that are known to co-assemble with and modify the gating properties of K-Channels. A total of 90 genes have Ensembl models that are correctly annotated as 1-to-1 K-Channel orthologs in the zebra finch genome compared to humans. An additional 16 Ensembl models were related to K-Channels but had incorrect annotations (Table 2A). These are misidentified as pseudogenes or novel genes, have an incorrect orthology annotation, represent different portions of the same genes, or represent alleles. Correction of these errors resulted in the identification of 8 additional 1-to-1 orthologs compared to humans, and 5 additional K-Channel genes that are absent in humans. We also identified 4 K-Channel genes for which there are currently no predictive Ensembl models in zebra finches (Table 2B), consisting of 2 additional 1-to-1 orthologs and 2 more K-Channel genes that are absent in humans. Together with the 90 initial orthologs, these additional 17 genes account for 107 K-Channel genes in the zebra finch genome, 100 being 1-to-1 orthologs of human genes.

**Table 1:** Potassium channels genes in the zebra finch genome

	**Revised HUGO term ∆**	**IUPHAR name (or other terms)**	**Ensembl model or chromosome location**	**Model**^**!**^	Chicken ortholog ensembl gene ID*	**dN/dS ratio vs. chicken ^Ω**	**Song system expression and marker**				**ESTIMA *In Situ *Clone genbank ID**
							**HVC**	**LMAN**	**RA**	**X**	
**6 TRANSMEMBRANE / 1 PORE K-CHANNELS**											
***KCNA Subunits - 6TM/1P (potassium voltage-gated channel subfamily a, shaker-related***											
	KCNA1	K_v_1.1	ENSTGUG00000011956	Complete	ENSGALG00000017280	0.01	**+++ (↑↑)**	**+++ (↑↑)**	**+**	**+++ (↑)**	FE737967
	KCNA2	K_v_1.2	ENSTGUG00000000897	Partial	ENSGALG00000000442	0.00	**+**	**+**	**+**	**+**	FE720882
	KCNA3	K_v_1.3	ENSTGUG00000000902	Partial	ENSGALG00000000447	0.01	**N.D.**	**N.D.**	**N.D.**	**N.D.**	No clone
	KCNA4	K_v_1.4	ENSTGUG00000004781	Complete	ENSGALG00000012142	0.04; D	**++ (↑)**	**+**	**o (↓)**	**+ (↓)**	DV952065
	KCNA5	K_v_1.5	ENSTGUG00000018536	Partial	ENSGALG00000017279	0.10	**N.D.**	**N.D.**	**N.D.**	**N.D.**	No clone
#	KCNA6	K_v_1.6	ENSTGUG00000011959	Partial	ENSGALG00000017281	0.04 (1); I,D	**+**	**+**	**+**	**+**	FE733881
	KCNA10	K_v_1.10	ENSTGUG00000000862	Complete	ENSGALG00000000441	0.03	**N.D.**	**N.D.**	**N.D.**	**N.D.**	No clone
***KCNA Beta Subunits (voltage-gated channel beta subunits)***											
#	KCNAB1	HKvβ3	ENSTGUG00000011240; ENSTGUG00000011253	Partial	ENSGALG00000010269	0.02 (2)	**++ (↑)**	**++ (↑)**	**++ (↑)**	**++ (↑)**	DV947734
$	KCNAB2	HK_v_β2	ENSTGUG00000002658	Complete	ENSGALG00000000912	0.07; I,D	**+ (↑↑)**	**++ (↑↑)**	**++ (↑)**	**+ (↑)**	DV957424
**KCNB Shab-related K-Channels and Accessory Subunits**											
***KCNB Subunits - 6TM/1P (voltage-gated channel subfamily b, shab-related)***											
	KCNB1	K_v_2.1	ENSTGUG00000008781	Partial	ENSGALG00000004758	< 0.01	**o**	**o**	**o**	**o**	FE732864
#	KCNB2	K_v_2.2	ENSTGUG00000011511	Partial	ENSGALG00000022800	0.09 (1); D	**N.D.**	**N.D.**	**N.D.**	**N.D.**	No clone
***KCNF Modulatory Subunits - 6TM/1P (voltage-gated channel subfamily f)***											
	KCNF1	K_v_5.1	ENSTGUG00000013065	Partial	ENSGALG00000016448	0.06	**+**	**o (↓)**	**o (↓)**	**+**	DV951251
***KCNG Modulatory Subunits - 6TM/1P (voltage-gated channel subfamily g)***											
	KCNG1	K_v_6.1	ENSTGUG00000008640	Complete	ENSGALG00000007980	0.03	**+ (↓)**	**++**	**++**	**+ (↓)**	CK301661
	KCNG2	K_v_6.2	ENSTGUG00000006664	Partial	ENSGALG00000012652	**0.11**	**N.D.**	**N.D.**	**N.D.**	**N.D.**	No clone^∑^
	KCNG3	K_v_6.3	ENSTGUG00000003439	Complete	ENSGALG00000009919	0.05; I	**No signal Detected**				CK313822
	KCNG4	K_v_6.4	ENSTGUG00000004343	Partial	ENSGALG00000005502; ENSGALG00000023534*	< 0.01 (1)	**N.D.**	**N.D.**	**N.D.**	**N.D.**	No clone
***KCNS Modulatory Subunits - 6TM/1P (voltage-gated subfamily s, delayed-rectifier)***											
	KCNS1	K_v_9.1	ENSTGUG00000005049	Partial	ENSGALG00000004074	0.01	**++ (↑)**	**+**	**+++ (↑↑)**	**++ (↑)**	FE729668
#	KCNS2	K_v_9.2	ENSTGUG00000012021	Partial	ENSGALG00000017690	0.03 (1)	**+ (↓)**	**+ (↓↓)**	**o (↓)**	**o**	CK307490
	KCNS3	K_v_9.3	ENSTGUG00000013126	Complete	ENSGALG00000016470	0.06	**+**	**+**	**+**	**+**	DV958330
***KCNV Modulatory Subunits - 6TM/1P (voltage-gated subfamily v)***											
	KCNV1	K_v_8.1	ENSTGUG00000012266	Complete	ENSGALG00000022481	0.07	**No signal Detected**				CK316773
	KCNV2	K_v_8.2	ENSTGUG00000005377	Partial	ENSGALG00000010173	0.02	**N.D.**	**N.D.**	**N.D.**	**N.D.**	No clone
#, ∆	KCNV2L		ENSTGUG00000000100	N/A	LOC100859639	0.04 (1, 2)	**N.D.**	**N.D.**	**N.D.**	**N.D.**	No clone
**KCNC Shaw-related K-Channels and Accessory Subunits**											
***KCNC Functional Subunits - 6TM/1P (voltage-gated subfamily c, shaw-related)***											
	KCNC1	K_v_3.1	ENSTGUG00000008821	Partial	ENSGALG00000006220	0.01	**++ (↑)**	**+ (↑)**	**+ (↑↑)**	**++**	CK302978
	KCNC2	K_v_3.2	ENSTGUG00000007354	Partial	ENSGALG00000010204*	0.01 (2)	**+ (↓)**	**++**	**+**	**+**	DV951094
	KCNC4	K_v_3.4	ENSTGUG00000000883	Partial	ENSGALG00000000400*	0.03 (2); D	**N.D.**	**N.D.**	**N.D.**	**N.D.**	CK308792†
**KCND Shab-related K-Channels and Accessory Subunits**											
***KCND Functional Subunits - 6TM/1P (voltage-gated subfamily d)***											
#	KCND2	K_v_4.2	ENSTGUG00000004691;ENSTGUG00000004681	Partial	ENSGALG00000009066; ENSGALG00000021899	0.03 (2)	**o (↓)**	**++**	**o (↓)**	**++**	DV956417
	KCND3	K_v_4.3	ENSTGUG00000013643	Partial	ENSGALG00000001512	0.04; I,D	**++**	**+ (↑)**	**+ (↓)**	**++**	CK316852
***KCND Modulatory Subunits (kv interacting protein, a-type channel modulatory protein)***											
	KCNIP1	KChIP1	ENSTGUG00000014900	Partial	ENSGALG00000002132	0.02	**++**	**+**	**+**	**+**	CK305253
	KCNIP2	KChIP2	ENSTGUG00000009911	Partial	ENSGALG00000007666	0.03 (2); I	**+**	**o (↓)**	**o (↓)**	**++ (↑)**	FE723960
	KCNIP4	KChIP4	ENSTGUG00000009548	Partial	ENSGALG00000014405	0.02	**++**	**++**	**+**	**+**	DV959885
**EAG, ERG, ELK K-Channels and Accessory Subunits**											
***KCNH Subunits - 6TM/1P (voltage-gated subfamily h; ether-a-gogo, ether-a-gogo-like or ether-a-gogo-related)***											
	KCNH1	K_v_10.1	ENSTGUG00000003202	Partial	ENSGALG00000009877	0.02; I	**N.D.**	**N.D.**	**N.D.**	**N.D.**	No clone
	KCNH4	K_v_10.2	ENSTGUG00000002564	Partial	ENSGALG00000003354	0.06	**N.D.**	**N.D.**	**N.D.**	**N.D.**	No clone
	KCNH5	K_v_11.2	ENSTGUG00000012967	Partial	ENSGALG00000011858	0.02	**N.D.**	**N.D.**	**N.D.**	**N.D.**	No clone^∑^
	KCNH6	K_v_11.3	ENSTGUG00000001965	Partial	ENSGALG00000000505	0.03	**N.D.**	**N.D.**	**N.D.**	**N.D.**	No clone
	KCNH7	K_v_12.1	ENSTGUG00000006950	Partial	ENSGALG00000011082	0.04; NC	**No signal Detected**				DV948210
	KCNH8	K_v_12.2	ENSTGUG00000003211	Partial	ENSGALG00000011262	0.01; S;C	**+**	**+**	**+**	**+**	DV957478
***KCNE (ISK) Accessory Subunits (MinK, MiRP) - 1TM (voltage-gated subfamily e)***											
	KCNE1	MinK	ENSTGUG00000016311	Complete	ENSGALG00000016012	0.01 (1)	**N.D.**	**N.D.**	**N.D.**	**N.D.**	No clone
#, ∆	KCNE1P	NA	No model	N/A	No Ortholog Found	N/A (3)	**N.D.**	**N.D.**	**N.D.**	**N.D.**	No clone
	KCNE3	MiRP2	ENSTGUG00000013455	Complete	ENSGALG00000022696	< 0.01 (1,2)	**No signal Detected**				FE736597
	KCNE4	MiRP3	ENSTGUG00000007898	Complete	ENSGALG00000021057	0.09	**N.D.**	**N.D.**	**N.D.**	**N.D.**	No clone
**KQT-Like K-Channels and Accessory Subunits**											
***KCNQ Subunits - 6TM/1P (voltage-gated subfamily kqt)***											
	KCNQ1	K_v_7.1	ENSTGUG00000009167	Partial	ENSGALG00000006472	0.09; S	**N.D.**	**N.D.**	**N.D.**	**N.D.**	No clone
*#, ∆*	KCNQ1L	K_v_7.1	ENSTGUG00000012696	Partial	chr1:72414886–72434322	N/A (3)	**No signal Detected**				FE721111
	KCNQ2	K_v_7.2	ENSTGUG00000007434	Partial	ENSGALG00000005822	0.02; S	**++**	**+**	**+**	**++ (↑)**	DV954380
	KCNQ3	K_v_7.3	ENSTGUG00000012545	Partial	ENSGALG00000016246	0.09	**++**	**+ (↓)**	**++**	**++ (↑)**	CK316820
*#*	KCNQ4	**K**_**v**_**7.4**	ENSTGUG00000017337	Partial	ENSGALG00000003200	0.07 (1)	**N.D.**	**N.D.**	**N.D.**	**N.D.**	No clone
	KCNQ5	K_v_7.5	ENSTGUG00000012688	Partial	ENSGALG00000015932	0.09	**++ (↑)**	**+**	**o (↓)**	**++**	CK310570
**BK, SK, IK, and Slack/Slick K-Channels and Accessory Subunits**											
***KCNMA Alpha Subunits (a.k.a. Maxi-K, Slo, BK) (calcium-activated potassium subfamily m, alpha subunits)***											
	KCNMA1	K_Ca_1.1/ Slo1	ENSTGUG00000006514	Partial	ENSGALG00000004980	0.02	**+**	**+**	**+**	**+**	DV954467
***KCNMB Beta subunit (a.k.a. Maxi-K, Slo, BK) (calcium-activated potassium subfamily m, beta subunits)***											
	KCNMB1	K_Ca1_β1	ENSTGUG00000014898	Complete	ENSGALG00000002118	**0.14**	**No signal Detected**				FE729268
	KCNMB2	K_Ca1_β2	ENSTGUG00000010767	Complete	ENSGALG00000017469	0.03	**N.D.**	**N.D.**	**N.D.**	**N.D.**	DV957683†
	KCNMB4	K_Ca1_β4	ENSTGUG00000007043	Complete	ENSGALG00000010044	0.05	**o**	**o**	**o**	**o**	FE738514
***KCNN Subunits (a.k.a. SK) (small conductance calcium-activated channel)***											
	KCNN1	K_Ca_2.1/ sk1	ENSTGUG00000014658	Partial	JH375632:3,234-4,841	0.01 (1,2)	**N.D.**	**N.D.**	**N.D.**	**N.D.**	No clone
	KCNN2	K_Ca_2.2/ sk2	ENSTGUG00000001344	Partial	ENSGALG00000002539	0.04 (2)	**N.D.**	**N.D.**	**N.D.**	**N.D.**	No clone
	KCNN3	K_Ca_2.3/ sk3	ENSTGUG00000004125	Partial	LOC777372	< 0.01 (1,2)	**No signal Detected**				CK310929
***KCNT Subunits (a.k.a. SLACK and SLICK) (subfamily t)***											
	KCNT1	K_Ca_4.1/ slack	ENSTGUG00000006452	Complete	ENSGALG00000001645	0.02	**++**	**++**	**++ (↓)**	**+**	FE727550
	KCNT2	K_Ca_4.2/ slick	ENSTGUG00000004186	Partial	ENSGALG00000002451	0.04; I,D	**+**	**+**	**o (↓)**	**+++ (↑↑)**	CK310556
**IRK, GIRK, and Related K-Channels and Accessory Subunits**											
***KCNJ Subunits (inward rectifying IRKs, GIRKs, and related)***											
	KCNJ1	K_ir_1.1	ENSTGUG00000000587	Partial	ENSGALG00000001167	0.01	**N.D.**	**N.D.**	**N.D.**	**N.D.**	No clone
	KCNJ2	K_ir_2.1/ irk1	ENSTGUG00000002878	Complete	ENSGALG00000004376	0.01	**N.D.**	**N.D.**	**N.D.**	**N.D.**	FE729566†
#	KCNJ3	K_ir_3.1/ girk1	ENSTGUG00000012153	Partial	ENSGALG00000012537*	0.01	**++**	**++ (↑↑)**	**++**	**++ (↑)**	DV950153
#, ∆	KCNJ3L		ENSTGUG00000002970	N/A	ENSGALG00000009935*	0.03	**+++**	**+++**	**+++**	**+++**	FE722554
	KCNJ4	K_ir_2.3/ irk3	ENSTGUG00000010412	Complete	ENSGALG00000012254	0.01	**N.D.**	**N.D.**	**N.D.**	**N.D.**	CK302188†
	KCNJ5	K_ir_3.4/ girk4	ENSTGUG00000000586	Partial	ENSGALG00000001181	0.01	**+**	**+**	**+**	**+ (↑)**	CK315227
#, ∆	KCNJ5/9 L		Chr24:252787-253805	N/A	ENSGALG00000006922*	0.02 (1,2)	**N.D.**	**N.D.**	**N.D.**	**N.D.**	No clone
	KCNJ6	K_ir_3.2/ girk2	ENSTGUG00000004993	Complete	ENSGALG00000016054	0.01; S	**++**	**++**	**+ (↓)**	**+ (↑)**	FE720478
	KCNJ8	K_ir_6.1	ENSTGUG00000012087	Complete	ENSGALG00000013251	0.01; S	**No signal Detected**				CK312943
	KCNJ9	K_ir_3.3/ girk3	ENSTGUG00000015501	Partial	No ortholog found	N/A (3)	**N.D.**	**N.D.**	**N.D.**	**N.D.**	No clone^∑^
	KCNJ10	K_ir_4.1	ENSTGUG00000014138	Complete	LOC100857799	0.02 (1,2)	**N.D.**	**N.D.**	**N.D.**	**N.D.**	No clone
	KCNJ11	K_ir_6.2	ENSTGUG00000008677	Complete	ENSGALG00000020505	N/A (3)	**+**	**+**	**+**	**+**	FE729127
#	KCNJ12	K_ir_2.2/ irk2	ENSTGUG00000007541	Partial	ENSGALG00000004721*	0.01 (1)	**N.D.**	**N.D.**	**N.D.**	**N.D.**	No clone
	KCNJ13	K_ir_7.1	ENSTGUG00000007303	Complete	ENSGALG00000001490	0.09	**N.D.**	**N.D.**	**N.D.**	**N.D.**	No clone
	KCNJ15	K_ir_4.2	ENSTGUG00000004997	Complete	ENSGALG00000016055	0.03	**N.D.**	**N.D.**	**N.D.**	**N.D.**	No clone
	KCNJ16	K_ir_5.1	ENSTGUG00000015743	Partial	ENSGALG00000004373	0.02; I	**N.D.**	**N.D.**	**N.D.**	**N.D.**	No clone
**4 TRANSMEMBRANE / 2 PORE K-CHANNELS**											
***TWIK (inward rectifying), TASK (acid-sensitive), TREK (outward rectifying), THIK (halothane-inhibited), TALK (alkaline ph-activated) K-Channels - 4TM/2P (subfamily k)***											
	KCNK1	K_2P_1.1/ twik1	ENSTGUG00000010202	Complete	ENSGALG00000011005	0.04	**+**	**+**	**+**	**+**	FE734366
	KCNK2	K_2P_2.1/ trek1	ENSTGUG00000002899	Partial	ENSGALG00000009687	0.03	**+**	**+**	**+**	**+++ (↑)**	DV947959
	KCNK5	K_2P_5.1/ task2	ENSTGUG00000007722	Complete	ENSGALG00000010065	**0.14**; D	**+**	**+**	**+**	**+**	DV953320
	KCNK9	K_2P_9.1/ task3	ENSTGUG00000012636	Complete	ENSGALG00000016193	0.01	**++ (↑)**	**+**	**+**	**+**	FE721255
	KCNK10	K_2P_10.1/ trek2	ENSTGUG00000012403	Complete	ENSGALG00000010598	**0.23;** NC	**o**	**o**	**+**	**o**	FE734560
	KCNK12	K_2P_12.1/ thik2	ENSTGUG00000005557	Partial	ENSGALG00000008960*	0.06	**+**	**+**	**o (↓)**	**o**	FE724926
	KCNK13	K_2P_13.1/ thik1	ENSTGUG00000012450	Partial	ENSGALG00000010672	0.06; I	**+**	**+**	**+**	**+**	FE731455
	KCNK15	K_2P_15.1/ task5	ENSTGUG00000005191	Partial	ENSGALG00000004149	< 0.01	**N.D.**	**N.D.**	**N.D.**	**N.D.**	DV948159†
#	KCNK16	K_2P_16.1/ talk1	Chr3:29820700-29833238	Partial	ENSGALG00000020049	**0.18** (1,2)	**++**	**++**	**++**	**++**	FE723105
#, ∆	KCNK16L		ENSTGUG00000013021	N/A	ENSGALG00000012021*	**0.12**; S; NC	**N.D.**	**N.D.**	**N.D.**	**N.D.**	No clone
	KCNK17	K_2P_17.1/ task4	ENSTGUG00000007716	Partial	ENSGALG00000010068	**0.24**	**N.D.**	**N.D.**	**N.D.**	**N.D.**	No clone^∑^
	KCNK18	K_2P_18.1/ tresk1	ENSTGUG00000011054	Complete	ENSGALG00000009265	**0.22** (1,2)**;** S	**N.D.**	**N.D.**	**N.D.**	**N.D.**	No clone
**BCNG (brain cyclic nucleotide gated)**											
***HCN (potassium/sodium hyperpolarization-activated cyclic nucleotide-gated channel)***											
	HCN1	HAC2	ENSTGUG00000002345	Partial	ENSGALG00000014875	0.05; D	**+**	**+**	**+**	**+**	DV957366
	HCN2	HAC1	ENSTGUG00000000617	Partial	ENSGALG00000001342*	< 0.01 (2)	**N.D.**	**N.D.**	**N.D.**	**N.D.**	No clone^∑^
	HCN3		ENSTGUG00000016537	Partial	AADN03012245:69–809; AADN03018895:429–1605.	< 0.01 (1, 2)	**N.D.**	**N.D.**	**N.D.**	**N.D.**	No clone
	HCN4		ENSTGUG00000004104	Complete	ENSGALG00000001764	N/A (3)	**N.D.**	**N.D.**	**N.D.**	**N.D.**	No clone
*Note: HCN1 and HCN4 are also known to interact with KCNE2*											
**K-CHANNEL TETRAMERIZATION (btb/poz domain proteins)**											
	KCTD1		ENSTGUG00000010606	Partial	ENSGALG00000015124	< 0.01	**N.D.**	**N.D.**	**N.D.**	**N.D.**	FE722502†
	KCTD2		ENSTGUG00000008750	Partial	ENSGALG00000007934	< 0.01	**++**	**++**	**+**	**++**	FE723833
	KCTD3		ENSTGUG00000002895	Partial	ENSGALG00000009678	0.01 (2)	**++**	**+**	**+ (↓)**	**++**	CK301828
#, $	KCTD4		ENSTGUG00000018368	Complete	ENSGALG00000016975	0.05; S	**++**	**+**	**++**	**+**	FE727651
	KCTD5		ENSTGUG00000009562	Partial	ENSGALG00000006423	0.04	**++**	**+++**	**++**	**+**	CK301677
	KCTD6		ENSTGUG00000009191	Complete	ENSGALG00000007111	< 0.01	**++ (↑)**	**+**	**+**	**++ (↑)**	DV956204
	KCTD7		ENSTGUG00000005372	Partial	ENSGALG00000002618	0.03 (1); S	**N.D.**	**N.D.**	**N.D.**	**N.D.**	No clone^∑^
	KCTD8		ENSTGUG00000008473	Partial	ENSGALG00000014225	0.03	**N.D.**	**N.D.**	**N.D.**	**N.D.**	FE738572†
	KCTD9		ENSTGUG00000004348	Complete	ENSGALG00000000275	0.03; S	**No signal Detected**				FE726911
	KCTD10		ENSTGUG00000007316	Complete	ENSGALG00000005138	< 0.01 (1)	**No signal Detected**				FE738173
	KCTD12		ENSTGUG00000012550	Partial	Chr1:1638205-1638379	< 0.01 (1)	**++ (↓)**	**+ (↓↓)**	**+ (↓↓)**	**+++ (↑)**	CK306254
#, ∆	KCTD12L		ENSTGUG00000005525	Complete	ENSGALG00000009628*	0.06; D	**o**	**o**	**o**	**o**	FE734190
	KCTD14		ENSTGUG00000013019	Partial	ENSGALG00000017266	0.09	**N.D.**	**N.D.**	**N.D.**	**N.D.**	No clone
	KCTD15		ENSTGUG00000009508	Partial	ENSGALG00000004907	0.03	**+**	**+**	**+**	**+**	CK309195
	KCTD16		ENSTGUG00000000129	Partial	ENSGALG00000012322	0.03	**+**	**+**	**+ (↓)**	**+**	FE739532
	KCTD17		ENSTGUG00000010672	Partial	ENSGALG00000012486	0.06	**++**	**++**	**+++**	**+**	CK313062
#	KCTD18		Chr7:22311560-22315242	Partial	ENSGALG00000008155	**0.41** (1,2)	**N.D.**	**N.D.**	**N.D.**	**N.D.**	No clone
	KCTD20		ENSTGUG00000000921	Complete	ENSGALG00000000511	0.07; I,D	**++**	**++**	**+ (↑)**	**+**	DV959510
	KCTD21		ENSTGUG00000013013	Complete	ENSGALG00000017268	0.06	**++**	**++**	**++**	**++**	FE733511
	KCNRG		ENSTGUG00000012112	Partial	ENSGALG00000017012	**0.18**	**N.D.**	**N.D.**	**N.D.**	**N.D.**	No clone

# - Indicates a change/correction or addition to the zebra finch Ensembl Gene Model or Genebuild (see Table 2).

∆ - Gene is absent in humans; our terminology is based on modified HUGO terms.

$ - A related Ensembl model has been modified/corrected (see Table 2).

! - Partial models lack one or several exons present in human orthologs due to low sequence quality or genomic gaps.

* - Change/Correction to Chicken Ensembl Gene Model.

^ - Genes in bold/underlined have dN/dS values > 2X Average for K-Channel set (see Additional file 1: Table S5).

Ω - Amino acid change codes (S = Synonymous; C = Conservative; NC = Nonconservative; see Additional file 1: Table S3) or Indels compared to chicken codes (I = Insertion; D = Deletion; See Additional file 1: Table S4).

∑ − Evidence of brain expression based on cDNA/EST alignment. Clone not available for in situ hybridization.

† − Evidence of brain expression based on cDNA/EST alignment. Clone did not grow. ISH no completed.

*ND* No Data.

**Table 2:** Ensembl gene model corrections (A) and novel additions (B) to Ensembl Genebuild (e70)

**Revised HUGO term**	**Ensembl model or chromosome location**	**Current ensembl annotation (taegut3.2.4 )**	**Implication for zebra finch genome**	**Explanation**
**2A - Corrections**				
KCNA6	ENSTGUG00000011959	Novel Pseudogene	KCNA6 is present	Model truncated due to gaps in genomic sequence
KCNAB1	ENSTGUG00000011253; ENSTGUG00000011240	Novel Gene	KCNAB1 is present	ENSTGUG00000011253, which corresponds to the 3′ part of the gene, is incorrectly placed upstream of ENSTGUG00000011240, which represents the 5′ part of the same gene
KCNAB2 allele^$^	ENSTGUG00000013969	Novel Gene	KCNAB2 has an allele.	Model maps to Chr Unknown and has high sequence identity to KCNAB2, so it is likely an allele
KCNB2	ENSTGUG00000011511	Novel Pseudogene	KCNB2 is present	Model truncated due to gaps in genomic sequence
KCND2	ENSTGUG00000004681	Novel Gene	KCND2 is present	ENSTGUG00000004681 and ENSTGUG00000004691 (Table 1) are different parts of KCND2.
KCTD4	ENSTGUG00000018368	B5FX54_TAEGU	KCTD4 is present	Model truncated due to gaps in genomic sequence
KCTD4 allele^$^	ENSTGUG00000018204	B5FX54_TAEGU	KCTD4 is present (ENSTGUG00000018368; see above) but not duplicated	Model maps to Chr Unknown and has high sequence identity to KCTD4, so it is likely an allele
KCNJ3	ENSTGUG00000012153	Novel Gene	KCNJ3 is present; KCNJ3L is a duplication of KCNJ3.	Model not annotated
KCNJ3L	ENSTGUG00000002970	KCNJ3	Duplication of KCNJ3 (ENSTGUG00000012153), absent in humans	See results text
KCNJ12	ENSTGUG00000007541	Novel Pseudogene	KCNJ12 is present	Model truncated due to gaps in genomic sequence
KCNK16L	ENSTGUG00000013021	Novel Gene	Duplication of KCNK16 (no model), absent in humans.	See results text
KCNQ1L	ENSTGUG00000012696	Novel Gene	Duplication of KCNQ1 (ENSTGUG00000009167), absent in mammals	Model not annotated
KCNQ4	ENSTGUG00000017337	Novel Gene	KCNQ4 is present	Model not annotated
KCNS2	ENSTGUG00000012021	Novel Pseudogene	KCNS2 is present	Model truncated due to gaps in genomic sequence
KCNV2L	ENSTGUG00000000100	Novel Gene	Duplication of KCNV2 (ENSTGUG00000005377), absent in humans	See results text
KCTD12L	ENSTGUG00000005525	Novel Gene	Duplication of KCTD12 (ENSTGUG00000008750); absent in humans	See results text
**2B - Additions**				
KCNE1P	Chr1B_rand:70101-70314	None	Pseudogene duplication of KCNE1 (ENSTGUG00000016311), unique to zebra finch	Gene truncated but no gaps, high quality sequence
KCNJ5/9 L	Chr24:252787-253805	None	KCNJ5 or KCNJ9 duplicated, but absent in humans	Gene truncated due to gaps in genomic sequence
KCNK16	Chr3:29820700-29833238	None	KCNK16 is present	Gene truncated due to gaps in genomic sequence
KCTD18	Chr7:22280633-22353729	None	KCTD18 is present	Gene truncated due to gaps in genomic sequence

$ - These corrected models represent putative alleles and are therefore not included in Table 1.

### Novel K-Channel genes that are absent in humans

Seven of the K-Channel genes identified in the zebra finch genome (indicated by Δ on Table 1) are not defined by HGNC, and could not be found by BLAT alignments of predicted mRNA or protein sequences in the human genome. As detailed below (see Additional file 1: Table S1, and Additional files 2, 3, 4, 5 and 6), these genes are to some extent present in avian and/or other vertebrate organisms:

#### KCNJ3L

**Figure 2:**
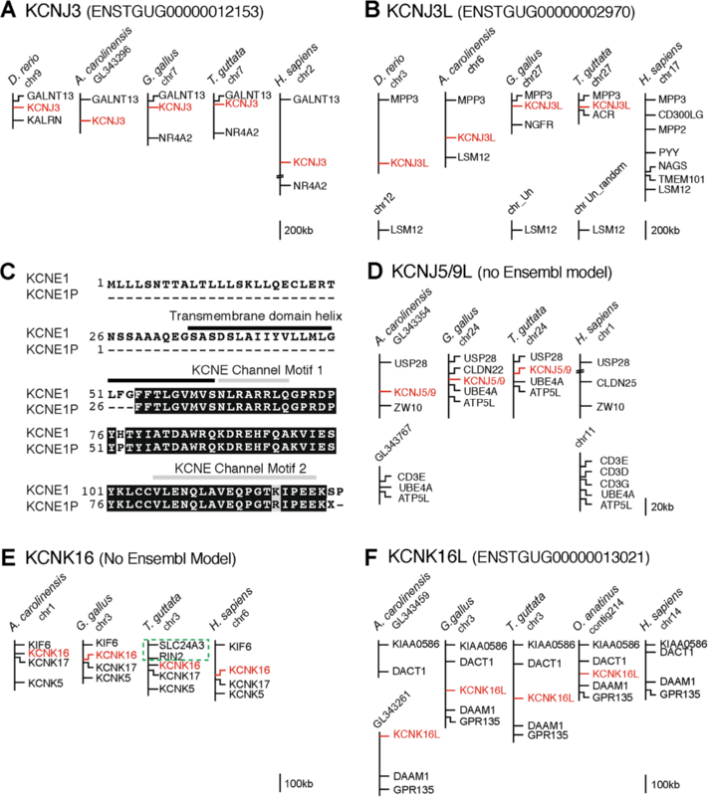
**Syntenic analysis of novel K-Channel genes (*KCNJ3L*, *KCNE1P*, and *KCNK16L*) that are absent in humans. (A**,**B** and **D**-**F)** Schematic representation of conserved chromosomal loci in select vertebrate species that contain orthologs of *KCNJ3***(A)**, *KCNJ3L***(B)**, *KCNJ5/9 L***(D)**, *KCNK16***(E)**, and *KCNK16L***(F)**. See Results text for details. The relative position of each K-Channel gene of interest is indicated in red; intervening genes are indicated in black. The chromosome or scaffold is indicated beneath each species name. Scale bars indicate the approximate distance between genes. **(C)** Alignment of predicted amino acid (AA) sequences for zebra finch *KCNE1* and *KCNE1P*. The numbers on the left indicate the relative position of AA residues in each sequence; residues shaded in black are identical, those in gray denote a conservative substitution. The position of highly conserved K-Channel transmembrane domain helices, as well as KCNE Channel signature motifs are indicated in black and grey, respectively.

Based on sequence similarity and synteny, partial model ENSTGUG00000012153 (on chr7, and flanked by *GALNT13* and *NR4A2*) is the likely zebra finch *KCNJ3* ortholog (synteny shown in Figure 2A). The predicted protein for ENSTGUG00000012153 aligns to a second locus on chr27 of zebra finch containing ENSTGUG00000002970, annotated as encoding an unidentified protein. This model is most similar to *KCNJ3* based on protein-protein alignment (57.2% similarity at the amino acid level; see Additional file 2), protein BLAST searches of GenBank databases, and BLAT alignments against other avian genomes. ENSTGUG00000002970 also contains signature motifs of the KCNJ sub-family, as well as additional motifs specific to *KCNJ3*. Of note, the 5′ end of *KCNJ3* itself is missing in zebra finch, likely due to a gap in genomic sequence. BLAT alignments and synteny analysis (Figure 2B) indicate that an ENSTGUG00000002970 ortholog is also present in chicken (ENSGALG00000009935; annotated as known, unidentified protein), lizard (ENSACAG00000004639; annotated as novel) and fish (ENSDARG00000059747; annotated as known, unidentified protein). We found the flanking loci (i.e. *MPP3* and *LSM12*) in human and mouse, but no evidence of a K-Channel-related ortholog. Thus, this gene is likely a duplication of *KCNJ3* that occurred in teleostomi, but was lost in mammals. We have therefore named this gene KCNJ3-Like (*KCNJ3L*).

#### KCNE1P

The 5′ portion of ENSTGUG00000016311, the zebra finch *KCNE1* ortholog, aligns secondarily to a region ~1 kb downstream of the primary *KCNE1* locus on chr1B_random of zebra finch. This second locus is not separated from *KCNE1* by any gaps in the genomic sequence, has good sequence quality, and shares 97.2% similarity with *KCNE1* at the amino acid level, but is not predicted by Ensembl. The predicted peptide lacks a start codon as well as a portion of the transmembrane domain predicted for *KCNE1*, but does contain two conserved signature domains for KCNE-type channels (Figure 2C). No transcripts are associated with this locus and there is no evidence of a comparable locus in the corresponding regions of the chicken and human genomes, both of high sequence quality, and without gaps. Thus, since this locus likely represents a novel pseudogene in zebra finch that resulted from a duplication of *KCNE1*, we have named it KCNE1-Pseudogene (*KCNE1P*). We note that this region is flanked by long terminal repeats (LTRs) and other related repeat elements, suggesting that this gene may have resulted from a retrotransposon-related duplication.

#### KCNJ5/9 L

Zebra finch *KCNJ5* and *KCNJ9*, and to a lesser extent *KCNJ3,* additionally align to an alternate zebra finch locus (chr24: 252,787 - 253,805) that is flanked by *USP28* and *UBE4A/ATP5L* and has no Ensembl predictions (Figure 2D). We have identified orthologs in chicken (ENSGALG00000006922, partial, annotated as LOC428244), lizard (ENSACAG00000009067, novel gene) and frog (ENSXETG00000022975, uncharacterized protein). There is also evidence for an orthologous locus in fish, but the search is complicated by gaps and chromosomal rearrangements. In mouse, *UBE4A* is non-syntenic with *USP28*, possibly resulting from an intrachromosomal rearrangement. Similarly, the human orthologs of *UBE4A* and *USP28* are rearranged compared to birds, and are found on different chromosomes (Figure 2D). In both cases, there is no evidence of any K-Channel-related orthologs. The chicken ortholog aligns entirely to the corresponding locus in zebra finch, suggesting that the finch ortholog is also complete, however evidence for mRNA expression is only present in chicken. The predicted peptide in zebra finch is conserved compared to chicken and lizard (83.5% and 78.5% identity respectively; see Additional file 3), with the exception of a 145 AA chicken-specific insertion (chicken residues 22–167) and a 30 AA finch-specific insertion (finch residues 291–320), the latter partially altering a protein motif that is specific to inward-rectifying K-Channels. The predicted proteins in all three species contain the 6-element protein fingerprint that is present in members of the KCNJ inward rectifier K-channel sub-family (PRINTS; [36]). Additionally, we found 2/5 motifs present in KNCJ9 orthologs, but found that the remaining 3/5 motifs for KCNJ9, as well as 6/6 motifs for KCNJ5, are predicted to be in protein regions that were included in the partial finch model prediction. Low quality sequence and genomic gaps likely prevented the prediction of a more complete model. Since this gene had the highest alignment scores to the genomic loci that contained KCNJ5 or KCNJ9, we conclude that this gene is likely a duplication of *KCNJ9* or *KCNJ5* that possibly occurred in euteleostomi, but was lost in mammals, and we propose naming it KCNJ5/9-Like (*KCNJ5/9 L*).

#### KCNK16L

ENSTGUG00000013021 on chr5 (annotated as *KCNK16*) aligns to a secondary locus (chr3: 29,820,700 - 29,833,238) where no gene model is predicted. BLAT alignments and partial synteny (Figure 2E) define corresponding loci in lizard, chicken, and human, which are also annotated as *KCNK16*. Thus, the secondary alignment of ENSTGUG00000013021 on chr3 appears to identify the true zebra finch ortholog of *KCNK16*. Due to an apparent chromosomal rearrangement, the genes immediately upstream of *KCNK16* in zebra finch (*RIN2* and *SLC24A3*; dotted box in Figure 2E) are different from those found in lizard, chicken, and human, where *KIF6* is present. Of note, this rearrangement in zebra finch may have important implications for the *KCNK16* promoter. ENSTGUG00000013021 itself appears to be a duplicate of *KCNK16* that is orthologous to genes found in chicken (ENSGALG00000012021), lizard (no model; chrUn_GL343261: 1,882,731 - 1,899,265), and frog (ENSXETG00000011960), each annotated as novel, hypothetical protein, or protein fragment (Figure 2F). Among mammals, orthologs were found in platypus (ENSOANG00000011839), horse (ENSECAG00000009184; not shown) and cow (ENSBTAG00000045799; not shown), but were notably absent in rodents and primates, including humans. We also were unable to find an ortholog in fish. A fingerprint scan analysis of the predicted proteins identified 2/2 motifs that define the KCNK sub-family of two-pore channels, and 3/3 motifs defining the 5 members of the TASK channel sub-family (KCNK3, KCNK5, KCNK6, KCNK15, and KCNK17) in lizard, chicken, and finch. In contrast, only 2/3 motifs were found in platypus, and none were found in horse and cow (Additional file 4). We conclude that ENSTGUG00000013021 is likely a duplication of *KCNK16* with TASK channel-like features, which was likely first duplicated in frogs and then lost in the clade containing the supraprimates (i.e. rodents, primates, and their allies). We have named it KCNK16-Like (*KCNK16L*).

#### KCNV2L

**Figure 3:**
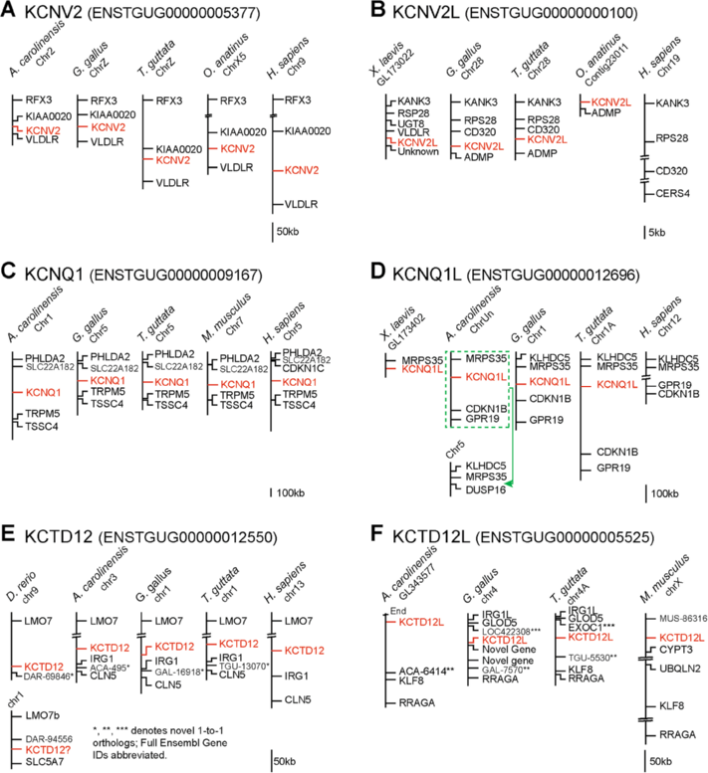
**Syntenic analysis of novel K-Channel genes (*KCNV2L*, *KCNQ1L*, and *KCTD12L*) that are absent in humans. (A**-**F)** Schematic representation of conserved chromosomal loci in select vertebrate species that contain orthologs of *KCNV2***(A)**, *KCNV2L***(B)**, *KCNQ1***(C)**, *KCNQ1L***(D)**, *KCTD12***(E)** and *KCTD12L***(F)**. See Results text for details. The green box in D highlights the placement of the conserved syntenic group in lizard on Chr_Un. Misplacement was likely caused by a genome assembly error. The relative position of each K-Channel gene of interest is indicated in red; intervening genes are indicated in black. The chromosome or scaffold is indicated beneath each species name. Scale bars indicate the approximate distance between genes.

ENSTGUG00000005377, the zebra finch ortholog of *KCNV2* (Figure 3A), aligns secondarily to a locus on chr28 of zebra finch that contains ENSTGUG00000000100 (unannotated). The predicted protein of ENSTGUG00000000100 lacks a start codon, but the prediction can be extended based upon an NSCAN prediction (chr28.016.a) to include a putative methionine. We have identified likely orthologs (Figure 3B) in chicken (Galgal4; chr28: 897,042-899,032, syntenic with ADMP/CD320 as in finch), frog (ENSXETG00000021930, syntenic with *RPS28*/*KANK3* as in finch), and platypus (ENSOANG00000002981, syntenic with *ADMP* as in finch), but not in other mammals, or in lizard, where the syntenic region is missing altogether due to incompleteness of the genome. We note that *ADMP* also appears to be missing in other mammals. The models in platypus (ENSOANG00000002981) and frog (ENSXETG00000021930), which are annotated as novel proteins, show 69.8% and 67.3% amino acid similarity with zebra finch, respectively (Additional file 5). Predicted proteins in all species analyzed contain 6/7 motifs conserved in KCNV channels (i.e. Kv9) as well as 8/8 protein motifs predicted for voltage-gated KCN channels. By comparison, the ortholog of *KCNV2* in frog is structurally similar to *KCNV2L*, possessing 6/7 KCNV and 8/8 KCN conserved motifs (not shown). We conclude that ENSTGUG00000000100 is likely a duplication of *KCNV2* in tetrapods, which was retained in monotremes and, along with *ADMP*, lost in other mammals. We have named this gene KCNV2-Like (*KCNV2L*).

#### KCNQ1L

ENSTGUG00000009167 (annotated as *KCNQ1*) BLAT aligns to two loci in the zebra finch genome. The primary locus, on chr5 and flanked by *TRPM5* and *SLC22A18*, appears to be the finch KCNQ1 ortholog as it shares synteny with chicken (ENSGALG00000006472), lizard (ENSACAG00000007664), fish (ENSDARG0000091599), and human (ENSG00000053918) orthologs (Figure 3C). The secondary locus on chr1A of zebra finch contains an unannotated model (ENSTGUG00000012696), is flanked by *MSPR35* and *CDKN1B*, and appears to be conserved in chicken (chr1: 72,414,890 - 72,415,036; no model due to gaps), lizard (ENSACAG00000004298; green box indicates a likely assembly error partially placing the syntenic group on Chr_Un), and fish (ENSDARG00000088397), but is absent in mouse and human (*MSPR35* and *CDKN1B* flank an apparent breakpoint; Figure 3D represents the two segments of the human chr12 as connected but separated by a large distance). In frog, the likely ortholog ENSXETG00000034068 is partially syntenic (*MRPS35*). The models in frog (ENSXETG00000034068) and lizard (ENSACAG00000004298) show 78.2% and 77.3% amino acid similarity with zebra finch, respectively (Additional file 6), however we note that the model predicted in finch spans gaps and is located partially in a low quality sequence region. Predicted proteins in finch and frog (the model in lizard appears incomplete) possess 3/3 motifs that define KCNQ-type channels and 5/9 motifs near the carboxy-terminus that collectively define the core signature of the KCNQ1 sub-family. The 8^th^ motif is partial and the 9^th^ motif is missing, likely due to incomplete protein predictions, and motifs 2 and 4 may have diverged with possible implications for channel function, or more likely appear altered due to the low quality of sequence used for model prediction. By comparison, the finch ortholog of KCNQ1 possesses all three KCNQ motifs and 8/9 KCNQ1 motifs located near the carboxy-terminus (not shown). Thus, this gene (predicted by ENSTGUG00000012696) appears to be a duplication of *KCNQ1* that likely occurred in fish, but was lost in mammals possibly due to a chromosomal rearrangement. We have named it KCNQ1-Like (*KCNQ1L*).

#### KCTD12L

Two models, ENSTGUG00000012550 and ENSTGUG00000005525, are currently annotated as *KCTD12* in zebra finch. ENSTGUG00000012550 (flanked by *LMO7* and *IRG1*) is most likely the zebra finch *KCTD12* ortholog, sharing synteny with models in fish (ENSDAR00000053542; *KCTD12.2*), lizard (ENSACAG00000026026; *KCTD12*), and humans (ENSG00000178695; *KCTD12*) (Figure 3E). In chicken, ENSTGUG00000012550 aligns to a partial locus in the correct syntenic block, which is truncated, possibly due to a chromosomal rearrangement. Of note, there is an apparent chromosomal rearrangement in fish that resulted in a duplication of *KCTD12* (ENSDARG00000053542) and *LMO7* (ENSDAR00000053535) on chr1. In contrast, model ENSTGUG00000005525 shares synteny with models that are annotated as *KCTD12* in chicken (ENSGALG00000009628) and lizard (ENSACAG00000006433) (Figure 3F). We also found several loci in fish, and one locus in frog that share sequence similarity with ENSTGUG00000005525, but were unable to confirm orthology due to gaps in the flanking regions. Though we were unable to identify an ortholog in humans, ENSMUSG00000041633 in mouse (annotated as *KCTD12B*) shares sequence and synteny with ENSTGUG00000005525. We conclude that ENSTGUG00000012550 is the finch ortholog of *KCTD12* and that ENSTGUG00000005525 represents a duplication of *KCTD12* present in sauropsids and mammals, and possibly in fish and frog, but absent in humans. We suggest annotating the latter KCTD12-Like (*KCTD12L*).

### K-Channel genes missing from the zebra finch genome

For 23 human K-Channel genes we found no evidence of zebra finch orthologs, nor did these yield hits in blastn searches of zebra finch EST/cDNA databases or tblastn searches of translated peptide databases using query sequences from chicken, lizard, frog, fish and representative mammals. Three of the missing genes (*KCNMB3L*, *KCTD9P1* and *KCTD9P2*) are recognized as human pseudogenes, the remaining 20 can be classified as follows (Additional file 1: Table S2):

a) Missing in zebra finch: 8 genes are absent in zebra finch, thus possibly in songbirds, but present in chicken. For *KCNE1L*, on chr4 flanked by *ACXL4* and *NTX2* in chicken, there are no sequence gaps at the corresponding region in zebra finches (on chr4A). Thus this gene is clearly missing in zebra finches. As for the other genes, gaps in the genomic sequence at the syntenic locations limit any firm conclusions about gene loss; in some cases the flanking genes in humans are also missing in finches.

b) Missing in avian genomes only: 8 genes are absent in zebra finch, chicken or turkey genomes, but present in lizard. For most of these, one or more of the flanking genes in lizard, frog, fish and/or mammals are also absent in avian genomes. Since the absence of syntenic groups in both avian lineages is unlikely to be entirely explained by gaps in the genomic sequence, we conclude that these genes were probably lost in birds.

c) Missing in avian and lizard genomes only: 2 genes and their syntenic genes are not present in either birds or lizard, but could be identified in frog. Although we cannot exclude the possibility that orthologs might be present within genomic sequence gaps, it seems more parsimonious to conclude that they were lost in sauropsids.

d) Missing in avian, lizard and frog genomes: 2 genes could not be found in birds, lizard or frog, and thus either represent an even more basal loss in tetrapods, or genes that are unique to mammals or subgroups therein. In fish, genes annotated as *KCNK7* and *KCTD11* map to very different syntenic regions from mammals, and thus are likely to represent independent duplications of related paralogs.

### Allelic variants of K-Channel genes in zebra finches

Several K-Channel genes had secondary high-scoring alignments outside the main genomic assembly of zebra finch, thus potentially representing allelic variants (Additional file 1: Table S3). To identify variants that might affect peptide structure/function, we examined secondary alignments more closely by using only the predicted peptide of each K-Channel ortholog in zebra finch as queries in the BLAT searches. We only considered alignments to regions of high quality sequence and that had >95% identity to the query, ignoring cases where the Ensembl model itself was from a region of poor sequence quality (see Methods for details). This restrictive search may have missed variants, but it is unlikely that the variants detected represent sequence errors.

We identified 12 K-Channel genes had secondary alignments that meet the criteria above (indicated in Table 1 by letter-code abbreviations in dN/dS Ratio vs. Chicken column; see Additional file 1: Table S3 for details). Almost all of these alignments were present on a Chr_Unkown or, in rare cases on a known chromosome but within a short region flanked by gaps, making it unlikely they represent duplicated genes. They typically consisted of short aligned segments representing one to a few exons at 90-99% nucleotide identity with the Ensembl model and containing 1 (rarely 2–3) substitution(s) per exon. While the majority of substitutions were synonymous, one was conservative (a *KCNH8* variant had an S for T at residue 366, between two transmembrane domains within the ion transport region), and three non-conservative (a *KCNK10* variant had an A for T at residue 44, in a region that encodes a known functional domain; a *KCNH7* variant had a D for N at residue 882, close to the carboxy-end tail of the predicted peptide; and a *KCNK16L* variant had an S for Y at residue 78, within a predicted ion transport domain, and a deletion of an F).

### Unique features of protein coding domains of K-Channel genes in zebra finches

To identify insertions/deletions in K-Channel genes of zebra finches, we aligned the coding sequences of the zebra finch and chicken K-Channel orthologs using ClustalW and searched for segments that are absent in chicken or zebra finch sequences. We note that we excluded from this analysis cases where a given segment was misaligned due to a poor model prediction, predictions based on low quality sequence, and/or genomic gaps that affected protein prediction. We identified 17 genes containing features that appeared to be different between zebra finch and chicken (indicated in Table 1 by the letters “I” and “D” in dN/dS Ratio vs. chicken column; see Additional file 1: Table S4 for details). Although for the majority of genes (15/17) these features occurred within regions that contain no domain prediction, *KCNA4* has a finch specific deletion of ‘NG’ at position 681 that occurs within an ion transport domain, and *KCNA6* has three different 1–2 amino acid insertions and deletions that occur within a predicted tetramerization domain and the ion transport domain, thus possibly affecting these protein functions.

### K-Channel genes under high selective pressure in zebra finch

Some K-Channels have been suggested to be under high selective pressure in songbirds, based on dN/dS ratios in comparison with chicken [6,12]. To further determine whether additional K-Channel genes might also be under high selective pressure, we first compiled dN/dS ratios for all 100 K-Channel genes that had orthologs in chicken based on an Ensembl summary of gene orthologs. We then calculated the average value for this set of ortholog pairs (0.06) and identified 12 genes that had particularly high dN/dS ratios as compared to the group average (i.e. > 2X above average). However, careful analysis revealed that more than half of these high dN/dS genes (Additional file 1: Table S5A), including one gene previously reported as being under positive selection (e.g. *KCNC2*; [12]), had artificially high values resulting from improperly aligned protein coding models, incomplete model predictions, and/or models derived from low quality sequence. We therefore verified and recalculated dN/dS values as necessary for each gene using segments of high quality sequence and visual confirmation of protein alignments (see Methods). After establishing a revised average dN/dS (0.045) and a new cut-off value (> 0.1), we were able to confirm that 6 out of the original 12 genes (*KCNMB1*, *KCNK5*, *KCNK10*, *KCNK17*, *KCNK18*, and *KCNRG*), as well as 4 additional genes (*KCNG2*, KCNK16, *KCNK16L*, and *KCTD18*) have high dN/dS ratios as compared to the group (Table 1, bold/underlined in dN/dS Ratio vs. Chicken column, and Additional file 1: Table S5B). We note that these 10 cases cannot be explained by low sequence quality or genomic gaps. Out of these, 5 were found to have substitutions within regions with no predicted functional domains, while 5 others have substitutions within a predicted transmembrane domain (i.e. *KCNG2*, *KCNK16*, *KCNK16L*, *KCNK18*), an ion transport domain (i.e. *KCNK16*, *KCNK18*), and/or a tetramerization domain that facilitates alpha subunit assembly (*KCNRG*). To determine whether the 5 genes with changes that could affect channel function are diverging faster in chicken or finch, we next examined the dN/dS ratio values for chicken and finch versus lizard (or turtle, when lizard data was not available). We found that two genes (*KCNMB1* and *KCNK16*) appear to be more rapidly evolving in the zebra finch lineage, 2 genes (*KCTD18*, *KCNRG*) are evolving more rapidly in chicken, and 1 (*KCNK16L*) is more rapidly evolving in birds. While the implications of these changes in coding sequences are unclear, since these genes differ between chicken and finch it is not unreasonable to predict that they might be associated with specific passerine vs. galliform traits, including vocal-learning. While simplistic, the genomes of several avian species are currently being sequenced and should allow future investigators an opportunity to conduct a more detailed and precise analysis of genes that are under high selective in various avian lineages.

### Brain distribution and differential expression of K-Channel genes in the song system

Of the 107 genes we identified as K-Channel genes in zebra finches, we found direct evidence of brain expression for 76 (Table 1). Evidence primarily consisted of the presence of representative clones (cDNAs) from zebra finch brain transcriptome collections [37,38]. The 31 remaining genes are not represented in the current cDNA databases and thus may be expressed at low levels in the brain, and/or primarily expressed in non-neuronal tissues. *In situ* hybridization confirmed that ~85% (53 out of 63) genes for which we were able to obtain riboprobes are expressed in the brain. Since our main interest was the identification of K-Channels that may be involved in some aspect of singing or vocal plasticity, we focus our analysis here on genes that we found to be expressed in the major telencephalic song nuclei of adult males (i.e. HVC, RA, LMAN, and Area X; Figure 1). A more comprehensive report of the brain-wide expression of K-Channel genes in zebra finches will be presented elsewhere.

The vast majority of K-Channel genes detected by *in situ* (50/53) were found to be expressed in at least one of the major song control nuclei, at variable levels (Table 1; relative expression in different nuclei indicated by crosses in the respective columns). Thus, a large number of genes may be involved in the neural control of birdsong. More than half of these (29/55) were differentially expressed in one or more song nuclei relative to the surrounding areas, and most major K-Channel gene sub-families had at least one member that showed differential expression in the song system. These genes appear to represent a specific subset of regulatory targets that could influence song learning and vocal plasticity due to their differential expression in the anterior forebrain pathway (AFP), and/or contribute to the maintenance or production of learned song based on their differential expression in the direct motor pathway (DMP). A summary of their differential expression is presented in Table 1 (see arrows in the expression columns).

### K-Channel differential expression in the anterior forebrain pathway (AFP)

**Figure 4:**
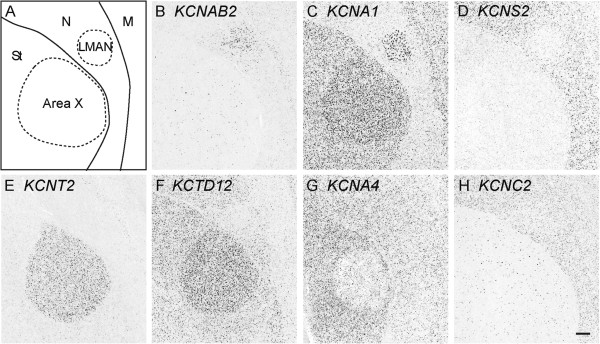
**Differential expression of K-Channel genes in the anterior forebrain pathway (LMAN and Area X) (A).** Camera lucida drawing of a parasagittal section (~1.4 mm from the midline) depicting anterior portions of the nidopallium and the medial striatum, including song nucleus LMAN and Area X (approximate location is indicated in Figure 1; laminae are depicted by thin lines; nuclei are indicated by dotted lines). **(B**-**H)** Representative photomicrographs of select K-Channel genes that are selectively expressed in LMAN and Area X. While some genes show almost exclusive expression in either LMAN **(B)** or Area X **(E)**, others show varying patterns of expression in both nuclei, including very specific cell populations. Scale bar: 200 μm. Gene abbreviations are given in Table 1. See Abbreviations for a complete list of anatomical abbreviations.

**Figure 5:**
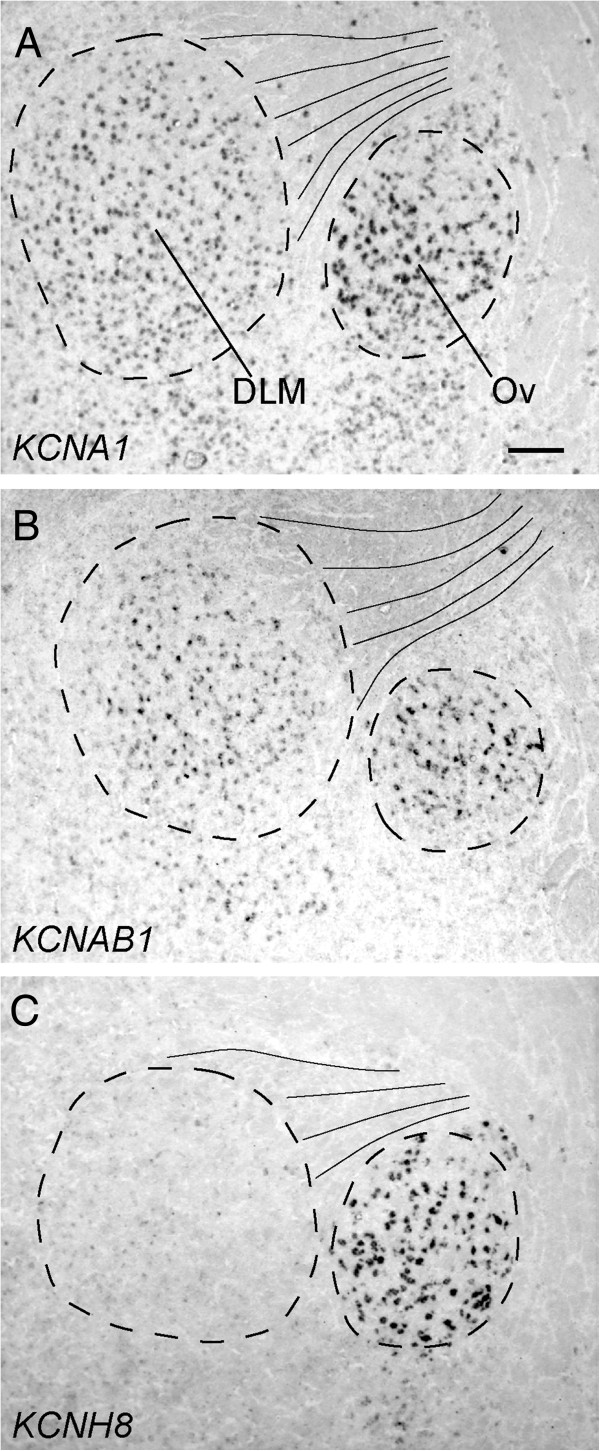
**Differential expression of K-Channel genes in the dorsal thalamus (DLM and Ov).** Photomicrographs of *in situ* hybridizations for KCNA1, KCNAB1, and KCNH8 taken in adjacent parasagittal sections at a brain level that includes song nucleus DLM, and auditory nucleus Ov (approximate location is indicated in Figure 1; dotted lines approximate nuclear boundaries based on dark field illumination; thin lines denote myelinated fibers). K-Channel genes reveal highly selective expression in DLM and Ov **(A** and **B)**, as well as Ov alone. **(C)**. Scale-bar: 100 μm. See Abbreviations for a complete list of anatomical abbreviations.

We identified 20 K-Channel genes with differential (either higher or lower) expression in LMAN and/or Area X compared to the surrounding brain region, including some of the most striking examples of differential expression in this gene family in the song system (Table 1, Figure 4). For example, *KCNAB2* (Figure 4B), which encodes a protein that modulates *KCNA1*, and *KCNA1* itself (Figure 4C), had high expression in LMAN, but relatively low expression in nidopallium. In contrast, *KCNS2* (Figure 4D), a potent modulator of the KCNB channel sub-family, showed a nearly opposite pattern of expression, and exhibited little or no expression in LMAN. Similarly, *KCNT2* and *KCTD12* showed elevated expression in Area X (Figure 4E, F), while *KCNA4* showed low expression in this nucleus, but strong expression in the surrounding striatum (Figure 4G). Some K-Channels had distributions suggestive of cell type specific regulation. *KCNC2* (Figure 4H), for example, which was not found to be differential in Area X or LMAN, and to some extent *KCNAB2*, were both found to label a sparse population of cells that are broadly distributed throughout the striatum, including Area X. Given this distribution, it seems reasonable to conclude that *KCNC2* and *KCNAB2* are expressed in a subset of striatal neurons. Although we did not conduct a systemic analysis of K-Channel expression in auditory brain areas, or in the medial part of the dorsolateral nucleus of the anterior thalamus (DLM), the thalamic nucleus that completes the cortico-striatal-thalamocortical AFB loop (Figure 1), we note that some genes were differentially expressed in these brain nuclei. For example, KCNA1 and one of its accessory subunits, KCNAB1 are strongly expressed in both DLM, and in an adjacent auditory nucleus, Ovoidalis (Ov; Figure 5A and B). Notably, while KCNA1 expressing cells are distributed throughout DLM, those expressing KCNA1B are more tightly clustered within the core region of DLM. In contrast, KCNH8, which shows little to no expression in DLM, is highly expressed in Ov (Figure 5C).

### K-Channel differential expression in the direct motor pathway

**Figure 6:**
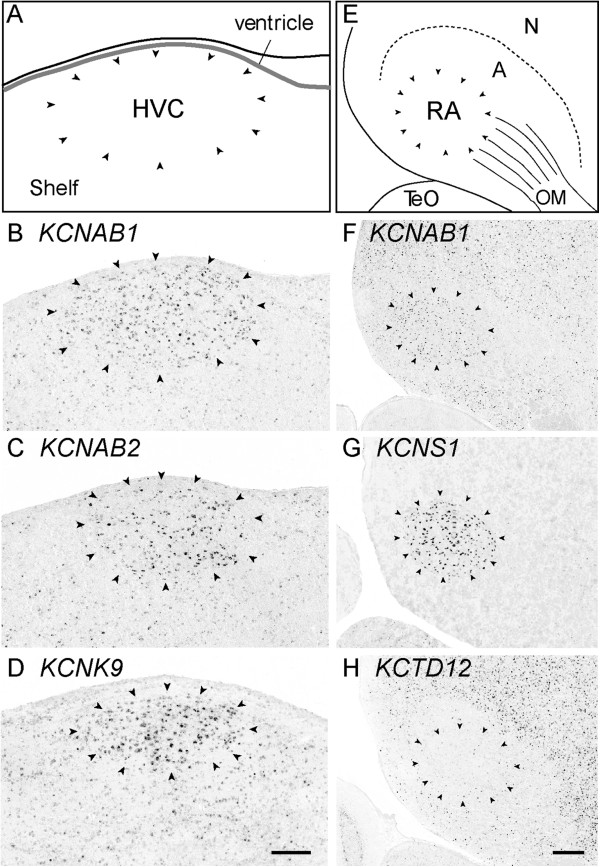
**Differential expression of K-Channel genes in the direct vocal-motor pathway (HVC and RA). (A**, **E)** Camera lucida drawings of a parasagittal section (~2.0 mm from the midline) depicting the dorso-caudal portion of the nidopallium, including song nucleus HVC (approximate location is indicated in Figure 1; HVC is indicated by arrowheads, ventricle is shaded in grey), and the ventro-caudal portion of the arcopallium and song nucleus RA. The location of a lamina is indicated by the dotted line; arrowheads delineate the approximate boundaries of HVC and RA; thin lines (in **E**) denote fibers of the occipitomesencephalic (OM) tract. *In situ* hybridization images for K-Channel genes reveal highly selective (positive or negative) markers of HVC **(B-D)** and RA **(F-H)**. Scale-bar: 200 μm. See Abbreviations for a complete list of anatomical abbreviations.

We identified 24 different K-Channel genes with higher or lower expression in HVC and/or RA as compared to their surrounding brain subdivisions (Table 1); typical examples are presented in Figure 6. Within HVC, a number of genes, including *KCNAB1*, *KCNAB2*, and *KCNK9* (Figure 6B-D) showed varying degrees of elevated expression. Similarly, in RA, genes such as *KCNAB1* (Figure 6F) and *KCNS1* (Figure 6G) showed markedly high expression, the latter providing a remarkably clear definition of the nuclear boundary of RA. Notably, the relative distributions and size of the neurons labeled by *KCNAB1* and *KCNS1* suggests that some genes may be specific markers of unique cell types in RA. In contrast, other K-Channels such as *KCTD12* were expressed within the arcopallium, but showed low or even absent expression in RA (Figure 6H).

### Members of the KCNA sub-family of K-Channels are differentially expressed in the song system

**Figure 7:**
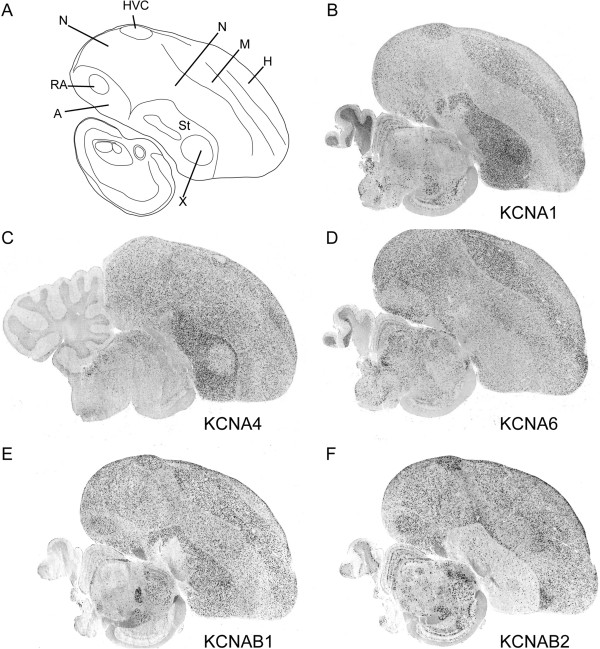
**Expression profiles for select members of the KCNA and KCNAB gene subfamilies in adult male zebra finch brain. (A)** Camera lucida drawing of a parasagittal section (~2.0 mm from the midline). **(B**-**F)** Representative photomicrographs of *in situ* hybridizations of KCNA **(B**-**D)** and KCNAB **(E**-**F)** subunits that were found to be differentially expressed in one or more nuclei of the song system. LMAN is not consistently present in these sections. See Abbreviations for a complete list of anatomical abbreviations.

K-Channel genes can be grouped into sub-families based on their phylogenetic relatedness [2]. Our analysis indicates that multiple members of the same sub-family can show differential overlapping and non-overlapping patterns of expression in the song system. One prominent example was the KCNA sub-family of delayed rectifier K-Channels, and their associated beta subunits (KCNAB). Out of the 4 *KCNA* and 2 *KCNAB* channels for which we were able to derive riboprobes, *KCNA1* showed strong enrichment in HVC (Figure 7B) and LMAN (Figure 4C), as well as weaker enrichment in Area X (Figure 7B; see also Figure 4C). In contrast, *KCNA2* and *KCNA6* were found to be expressed but not differential in the song system, whereas *KCNA4*, despite being strongly expressed in striatum, was expressed at very low levels in Area X (Figure 7C and 4G). *KCNAB1* and *KCNAB2*, genes which encode the two known accessory subunits of KCNA-type channels, both showed strong expression in the pallial song nuclei HVC, RA, and LMAN (a more medial brain section through LMAN is shown in Figure 4B), but very different patterns in the striatum and Area X. Specifically, *KCNAB1* was highly expressed throughout the striatum, including Area X, while *KCNAB2* expression was sparse, suggesting that it might be marker of a distinct striatal or possibly pallidal cell type.

### Evidence for the differential expression of duplicated genes in the songbird brain

**Figure 8:**
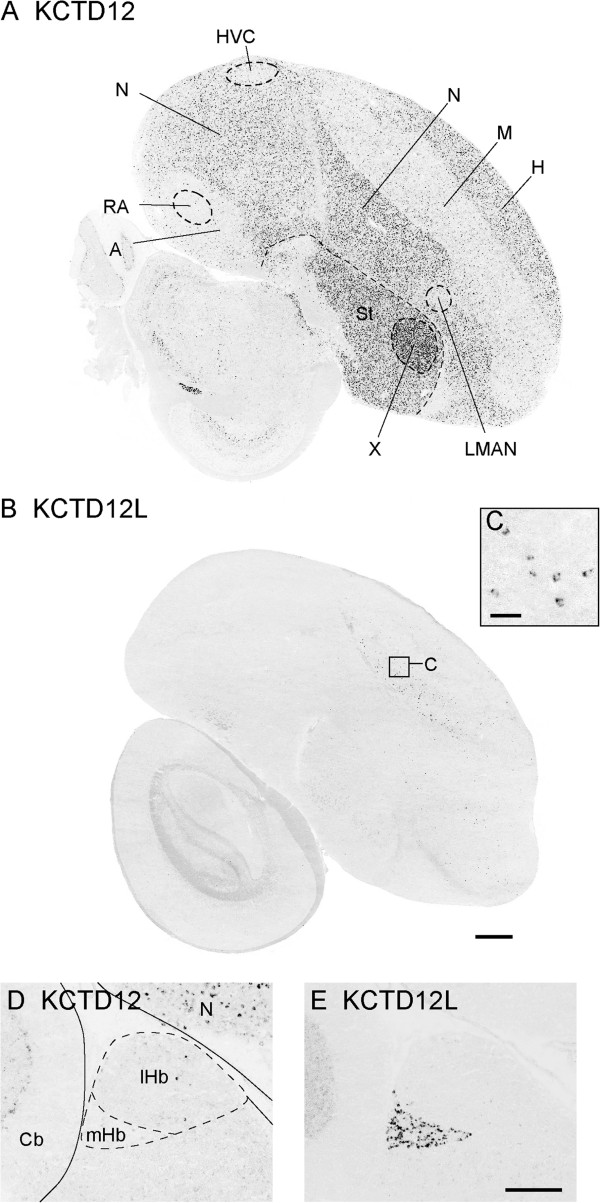
**Differential expression of *KCTD12 *and *KCTD12L *in adult male zebra finch brain. (A**-**B)** Photomicrographs of *in situ* hybridization for *KCTD12***(A)** and *KCTD12L***(B)**. While *KCTD12* is differentially expressed in specific nuclei of the song system, as well as other major brain subdivisions, *KCTD12L* shows relatively low expression throughout the brain with the exception of a specific cell type in the mesopallium. **(C)** High-power view of representative KCTD12L labeled cells in the mesopallium in a region indicated by the square in panel **B**. **(D**-**E)** High power views of KCTD12 and KCTD23L differential expression in habenula. While *KCTD12* shows little to no expression in the habenula **(D)**, *KCTD12L* labels a very specific population of cells in the medial, but not lateral portions of the habenula **(E)**. Scale bars: 500 μm in **A** and **B**; 50 μm in C; 200 μm in **D** and **E**. Gene abbreviations are given in Table 1. See Abbreviations for a complete list of anatomical abbreviations.

**Figure 9:**
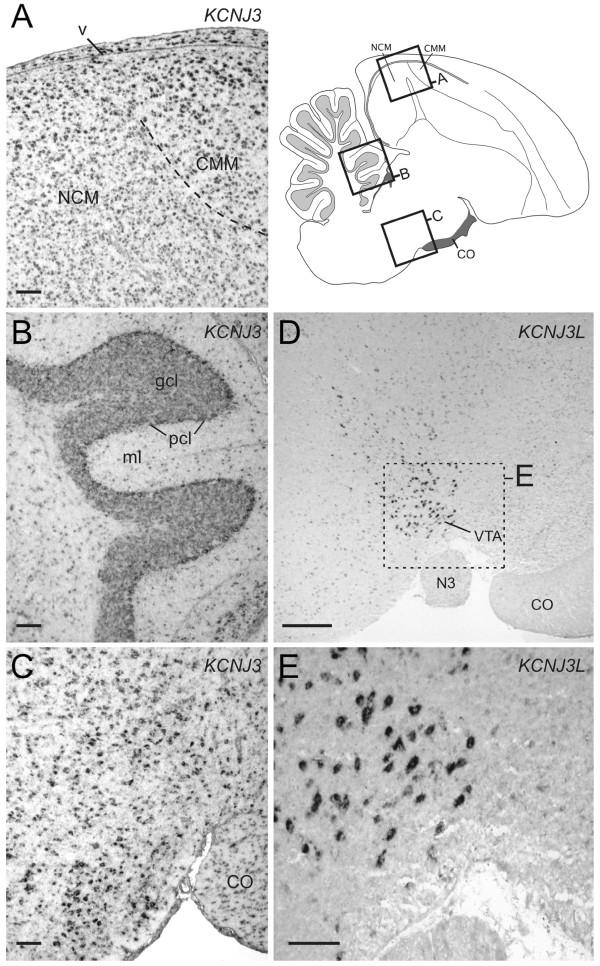
**Differential expression of *KCNJ3 *and *KCNJ3L *in adult male zebra finch brain. (A**-**E)** Photomicrographs of *in situ* hybridizations for *KCNJ3* and *KCNJ3L*. The approximately locations of the photomicrographs in **A**-**C** are depicted in the schematic to the right of panel **A**. The approximate location for the photomicrographs in panels **D** and **E** is similar to that for panel **C** except in a slightly more medial brain section. **(A**-**C)***KCNJ3* is highly expressed through the brain in most major cell types, including ependymal cells in the ventricle **(A)**, cells in the granular, Purkinje, and molecular layers of the cerebellum **(B)**, as well as various neuronal and glia populations in brainstem areas and fiber tracts **(C)**. **(D**-**E)** In contrast, *KCNJ3L* shows relatively low to no expression throughout the brain, but very strong expression in neurons of the AVT, and possibly substantia nigra **(D**; the dotted rectangle indicates the approximate position of the photomicrograph presented in **E)**. **(E)** High-power view of *KCNJ3L* labeled cells in AVT. Scale bars: 100 μm in **A**-**C** and **E**; 1 mm in **D**. Gene abbreviatio ns are given in Table 1. See Abbreviations for a list of anatomical abbreviations.

In a few cases, we found that a pair of duplicated genes had different patterns of expression in the songbird brain. For instance, *KCTD12* showed expression in most major brain subdivisions, but had low expression in pallial song nuclei (i.e. HVC, LMAN and RA), and high in striatal area X (Figure 8A). In contrast, *KCTD12L*, a likely duplication of *KCTD12* that is apparently missing in mammals, was barely detectable in most of the brain, but strongly expressed in a very discrete small cell type in the mesopallium (Figure 8B, C), as well as in the medial habenula (Figure 8E). *KCTD12* had only sparse labeling in lateral, and no expression in medial habenula (Figure 8D). *KCNJ3* showed expression throughout the brain, including in various neuronal and glial cell types in the ventricle, white matter, and fiber tracts (Figure 9A-C). In contrast, *KCNJ3L*, an apparent duplication of *KCNJ3* that is missing in mammals, was detectable at relatively low levels throughout the brain, but was highly enriched in the ventral tegmental area (VTA) and possibly part of substantia nigra (Figure 9D). These findings point to marked specializations between closely related genes (members of a duplicated gene pair) in terms of brain areas where they are active, and suggest corresponding differences in transcription regulatory domains within their promoters.

## Discussion

We have applied a comprehensive genome analysis strategy to identify the full complement of K-Channels and related genes in the zebra finch genome, and *in situ* hybridization to determine their brain expression, focusing on nuclei involved in birdsong production and learning. We chose humans as a starting point for K-Channel identification because they possess the largest and most completely described set of K-Channel genes. Furthermore, songbirds and humans share vocal learning, a rare trait that requires specialized vocal control brain structures that have been identified in both species. Our analysis revealed that a very large cohort of human K-Channel genes is also present in zebra finches, supporting a high degree of conservation across the two species, but we also identified several human genes that are missing in finches and some novel K-Channel genes that are present in finches but absent in humans, most of the latter representing conserved genes in non-mammalian vertebrates. Strikingly, nearly every major K-Channel sub-family (e.g. KCNA, KCNC, KCNQ, etc.) had at least one gene member that showed some degree of differential expression (higher or lower) within a song nucleus compared to its surround, supporting the notion that targeted gene regulation within this family may help shape the differential biophysical and excitable properties of vocal control areas in songbirds. Here we discuss the implications of our findings in the context of songbird biology and the evolution of circuitry for learned vocalizations.

### An improved approach for identifying orthologous gene sets

In spite of a completed draft genome assembly, well-curated sets of orthologous genes are not yet available in zebra finches. Our approach to identify the full complement of K-Channel genes in the current finch assembly consisted of first compiling a list of all protein coding genes recognized as K-Channels in humans, then BLAT-aligning the predicted protein sequences to the genome assemblies of zebra finches and other vertebrates of comparative interest, followed by orthology confirmation through synteny analysis. This approach allowed us to determine which subset of K-Channel orthologs in zebra finches are correctly annotated by Ensembl, but also to identify and correct several erroneously annotated models, and some relevant loci with no predicted models. Most of the latter are in regions of low quality sequence that also contain large or several sequence gaps, often interrupting open reading frames. Our analysis also helped to confirm and/or establish several gene gains or losses within the vertebrate phylogeny. We are confident to have identified the full complement of K-Channel genes in the current finch assembly, since we systematically re-BLAT-aligned every identified finch locus to the finch genome, to ensure no loci were being missed due to low cross-species sequence conservation. This was important as some unannotated loci could only be detected through alignments to other members of the same sub-family. We believe our approach provides a highly sensitive strategy for retrieving full ortholog sets, especially from low-coverage genomes.

### In search of finch-specific genomic features of K-Channel genes

The dramatic radiation of K-Channel genes in vertebrates, likely the result of ancient whole genome and chromosome duplications, as well as wholesale rearrangements [39], may have played a role in the rapid evolution of brain structures specialized for complex behaviors, including vocal learning. At the level of individual genes, changes affecting gene function may have included mutations, insertions and/or deletions in protein-coding sequences, changes in cis- and trans-regulatory domains, and even wholesale gene duplications/deletions. One of our goals was to determine whether the K-Channel gene family possesses features that are conserved in zebra finches (and by extension songbirds) and humans, thus pointing to possible physiological requirements associated with vocal production and learning. Specifically, we reasoned that we might find evidence for conserved K-Channel gene duplications/expansions, and/or protein coding insertions/deletions in zebra finches and humans, but absent in vocal non-learning mammals and birds (e.g. rodents, chicken).

Consistent with our hypothesis, we found that finches and humans share the vast majority of protein coding K-Channel genes, including representatives from each of the 21 major K-Channel sub-families (e.g., the 6TM and calcium-gated, the 2TM, the 4TM, the brain cyclic-nucleotide gated K-Channels, and the K-Channel tetramerization proteins). Furthermore, of the 23 human genes that appear to be missing in finches, 3 represent pseudogenes that likely do not influence cell physiology, and 7 may have been undetected in finches due to gaps in the current assembly. Thus out of 123 human K-Channel genes, only 13 (~10%) appear to be actually missing in songbirds, indicating a high degree of conservation in this gene family between these two greatly divergent species of vocal learners. Moreover, based upon consultation to the Allen Mouse Atlas of brain gene expression [40], 4 of these (*KCNA7*, *KCND1*, *KCNK6*, and *KCTD13*) do not appear to be expressed in the mammalian brain, 2 (*KCNAB3* and *KCNC3*) are widely expressed throughout the brain, suggesting a general functional role, and 3 (*KCNN4*, *KNCJ14*, and *KCNH3*) exhibit regionalized expression that largely overlaps with that of other members of these sub-families, suggesting functional redundancy in their brain distributions. Thus, it appears that the majority of genes that are missing in songbirds (or that are novel in mammals), are either not expressed in the brain, or have redundant functions with other family members that are present in birds.

Despite the apparent conservation among vocal learners, we also discovered that the complement of K-Channel genes is highly conserved between zebra finch and chicken, the latter species being incapable of vocal learning. For instance, we found just a single finch gene, *KCNE1P* that is absent in chicken, and since it is a pseudogene, it is not expected to influence neuronal excitability. In fact, nearly every novel K-Channel gene in finch that is absent in humans is present in chicken and/or other vertebrate taxa, suggesting that many of these genes represent relatively ancient gene duplications that were likely lost in some or in all mammals (Additional file 1: Table S1). Thus, it seems unlikely that K-Channel gene duplication events have played a prominent role in the evolution of neural circuitry for learned vocalizations. We next looked for evidence of protein coding insertions/deletions (indels) that might change the function of channels in zebra finches as compared to chickens. Intriguingly, we identified 16 K-Channel genes that contain finch-specific indels within their predicted protein coding domains. However, only two of these K-channels (*KCNA4* and *KCNA6*) had indels within a region containing a predicted functional domain, and in both cases this region is quite variable across taxa (e.g. mouse, lizard, human). Therefore, it seems unlikely that these amino acid substitutions are likely to affect channel function. Finally, our analysis was able to confirm and extend the observation that some K-Channel genes are under high selective pressure in finch compared to chicken [6,12]. We note that our analysis also revealed that nearly half of all K-Channel genes that could be inferred to have high dN/dS ratios based on Ensembl Compara are incorrect, and have artificially high dN/dS ratios that stem from improperly aligned protein coding models, incomplete model predictions, and/or models derived from low quality sequence. Interestingly, our analysis revealed that of the 10 genes with relatively high dN/dS ratios, 6 are members of the KCNK family of 2-TM channels, corresponding to half of all KCNK channels under high selective pressure in zebra finch. We note that this finding is not due to the disproportionate number of KCNK channels in the zebra finch genome, as the KCTD family, the largest family of K-Channel related genes, contains 20 members and just a single gene (*KCTD18*) under high selective pressure compared to chicken. While the implications of this finding are unclear, they do suggest that specific families of K-Channels may play a more prominent role in the evolution of zebra finches, and/or possibly the songbird lineage. It will be interesting to see if this remains the case for other members of the songbird clade as additional genome sequences become available (e.g. [41]). In any case, further analysis revealed just 5 cases where non-conservative amino acid substitutions might be expected to affect a predicted functional domain, and in just two cases (*KCNMB1* and *KCNK16*), we found that the dN/dS ratio comparing lizard vs. finch was higher than that of lizard vs. chicken, suggesting greater divergence in songbirds. Thus, overall, it appears that K-Channel genes are highly conserved among higher vertebrate lineages. It thus seems unlikely that the genomic features we have described for the K-Channel gene family in zebra finches, including novel genes or protein coding changes, are prominent mechanisms for tailoring the functional properties of circuitry for vocal production and maintenance.

As discussed next, we found strong evidence for the differential expression of K-Channel genes in song control nuclei. A comparable characterization of K-Channel expression in brain areas associated with speech and language (e.g. Broca’s, orofacial and laryngeal motor cortex, etc.) is lacking in humans, thus we cannot at present assess the degree of brain gene expression conservation across vocal learners. However, none of the detected song nuclei marker genes are specific to finches. These observations suggest that the specific properties conferred by K-Channel genes on vocal circuitry are most likely determined at the level of transcriptional regulation, pointing to promoter regulatory regions as critical elements to be examined in future studies.

### Functional tuning of brain circuits for the production and maintenance of learned vocalizations

Consistent with our expectations based on the function of K-Channels and their widespread expression in the mammalian brain [42-45], we found evidence for the expression of K-Channel genes encoding pore-forming (i.e., alpha) and accessory (i.e., beta) subunits in every major song nucleus in the adult zebra finch brain (Table 1). In fact, only 3 genes for which we had probes, and were able to detect signal, appear not to be expressed in the song system. Thus, it is possible that combinatorial patterns define the molecular constituents of K-Channels within song nuclei, forming a gene network that defines the homeostatic (e.g. membrane and resting potential) as well as dynamic states (e.g. firing rate, spike waveforms, and neurotransmission) of neurons that influence the maintenance and production of adult birdsong. However, we also found that more than half of all K-Channel genes (35/56) are differentially expressed (either up or down) in at least one vocal nucleus relative to its surrounding brain region. These genes constitute molecular markers of the song system, and they may underlie the unique electrophysiological features that define the properties of neurons in vocal nuclei. A deeper understanding of the roles that these K-Channel markers play in other systems may provide important insights into the cellular properties, firing behavior, and state dynamics of vocal control pathways.

### K-Channel specializations in an anterior forebrain pathway (AFP) for vocal plasticity

While lesions of AFP nuclei do not affect adult song *per se*, they do suppress song learning and reduce vocal plasticity, implicating the AFP as a key player in the sensorimotor learning and maintenance of birdsong. A major finding of the present study is the identification of a large number of K-Channel genes (21) that are specifically differentially expressed in the AFP (Table 1; Figure 4). The selective expression of these genes in the AFP suggests they may confer specialized electrophysiological features that are central for song learning and vocal plasticity.

Area X is located within the avian equivalent of the basal ganglia, but intriguingly it contains cells that present both striatal- and pallidal-like properties, based on anatomical, molecular, and neurophysiological criteria [46-51]. Area X receives primary input from LMAN, and in turn projects to DLM in the thalamus. While lesions of LMAN result in impoverished, but highly stereotyped adult song [16,17], partial lesions in Area X lead to abnormal, un-crystallized, and structurally variable song [18]. Thus, these nuclei serve functionally distinct roles during song learning. It seems reasonable to assume that these areas possess electrophysiological distinct properties, possibly related to K-Channel expression. Indeed, we found that 9 K-Channel genes are strong markers of Area X, 5 of LMAN, and 7 of both. Notably, *KCNAB2* (Figure 4A), *KCNA1* (Figure 4B), and *KCNT2* (Figure 4E) are among the strongest and most selective markers that we have found to date for LMAN and Area X, from a database of several hundred genes, hinting that these channels likely confer specific properties that are important for a functioning adult song system.

Some identified K-Channels markers of Area X have previously been linked to sensory and motor functions in mammalian systems. For example, *KCNT2*, which encodes a unique class of Na^+^-sensitive K-Channels (K_(Na+)_), is widely expressed throughout brainstem auditory nuclei in mammals, where it is thought to regulate the accuracy of neuronal spike timing [52]. Intriguingly, both singing- and auditory-related activity have been reported in Area X [49,51], suggesting that *KCNT2* might play a role in stabilizing, or locking-in, the fidelity of song-related firing patterns in Area X. Notably, Area X was also the only song nucleus showing higher differential levels of mRNA for a member of the KCNK sub-family of two-pore leak channels (i.e. *KCNK2*). This is intriguing as *KCNK2* is the only member of this sub-family that is capable of reversibly interconverting from an open leak channel to a voltage-dependent phenotype depending on the state of phosphorylation [53,54]. Its high expression in Area X suggests an underlying mechanism for regulating resting membrane potential, which would have important consequences for shaping cellular excitability [54,55]. Pharmacological and double-labeling studies will be needed to address what cell types these channels are expressed in, whether/how they may interact, and how the properties of individual neurons work together to establish the underlying properties of Area X.

Our *in situ* data also reveal that multiple members of the same K-Channel sub-family are co-expressed within the same nucleus, and possibly even cell type, suggesting that the combined properties conferred by these channels may be important for normal function. In Area X, two members of the G-protein coupled inward rectifying K-Channel (GIRK) sub-family (i.e. *KCNJ5* and *KCNJ6*) are highly expressed (Table 1). This is noteworthy, as GIRK channels are known to be activated by a variety of G-protein coupled receptors, including several shown to be expressed in Area X. For instance, D2 dopamine receptors are expressed in Area X [56] and are thought to be key players in the dopaminergic modulation of social-context dependent song learning [57-59]. Furthermore, D2 receptor activation dampens singing-related increases of *egr1* (*ZENK*) expression [59,60], and depresses evoked firing [58,61], responses that are consistent with the activation of IRK-type channels in other G-protein coupled pathways. We thus suggest that GIRK activation by D2 may represent a mechanism for modulating the properties of Area X neurons, a testable hypothesis that illustrates how molecular findings can help guide targeted pharmacological and electrophysiological studies.

LMAN’s core region contains magnocellular neurons that send dual projections to Area X and RA, as well as GABAergic neurons that influence the projection neurons through inhibition [62,63]. The magnocellular neurons are of particular interest since they provide direct input to the AFP via Area X, and the DMP via RA. Our analysis revealed that some K-Channel specializations distinguish LMAN from other song nuclei, including 1 of 32 of the KCNC sub-family members (*KCNC1*) for which we had probe. Interestingly, we found that *KCNC3*, which is prominently expressed in the brain of mammals, has been lost in birds, possibly amplifying the relative contributions of the remaining members of this channel sub-family. In mammals, KCNC-expressing neurons can be readily distinguished from other cells by their high-frequency firing, unique narrow spike waveform [64,65], and in some cases, expression of the calcium-binding protein parvalbumin. Intracellular recordings suggest that the fast gating kinetics conferred by KCNC channels are critical for maintaining high-frequency repetitive firing [66], leading some to argue that virtually every neuron that is capable of sustained firing likely expresses at least one KCNC type. While few intracellular studies have been conducted on the intrinsic properties of neurons in LMAN, the presence of parvalbumin expression in both parvocellular and magnocellular neurons (Lovell and Mello, unpublished observation) predicts that KCNC channels may define the fast-spiking properties of both cell types in LMAN.

### K-Channel specializations in a direct motor pathway (DMP) for singing

Lesions of HVC or RA abolish singing, indicating that the DMP provides the neural instructions or motor code for the production of learned song (Figure 1). While the exact nature of this code is unclear, the available evidence suggests a hierarchical organization with: (a) pre-motor HVC-to-RA neurons that burst sparsely during each song motif, thus apparently linked to the tempo of song, projecting to (b) RA neurons that fire repeatedly throughout the song, but in synchrony with temporal and spectral features of song syllables [67]. Given that these processes likely require very different cellular firing properties, we were not surprised to find major differences in K-Channel complements in HVC and RA. Indeed, out of the 27 K-Channel genes identified as markers of the DMP, 7 were exclusive to HVC, and 10 to RA.

We found that genes related to assembly of delayed rectifier voltage-gated channels are highly differential in HVC (Figure 6B), but generally weakly expressed or negatively differential in RA (Figure 6; *KCNA6*, *KCNA1*). This is intriguing as when KCNA subunits are paired with *KCNAB1* or *KCNAB2*, both of which are also elevated in HVC, the resulting inactivation gating properties become particularly suited for enhancing spike accommodation, broadening action potentials, and delaying spike after-hyperpolarizations [68,69]. HVC contains at least three distinct classes of neurons, namely neurons that project to Area X and RA (HVC_X_ and HVC_RA_, respectively), and local GABAergic interneurons (HVC_INT_), Importantly, HVC_RA_ neurons, the specific cell type that participates in pre-motor projection to vocal-motor nucleus RA, exhibit strong spike accommodation [30,31], a property that may underlie the sparse coding characteristic of this projection and that may be at least partially explained by the molecular profile we have found in HVC. We note the differential expression of KCNA channel subunits has been associated with the production of specific motor patterns in other systems. For example, in the lobster stomatogastric system, variation in K-current properties of pyloric neurons, which result from the differential expression of KCNA and KCNB subunits, has been shown to determine the firing order and phase relationships between circuit components, thus leading to the production of different motor patterns ([70,71]). In the song system, future double-labeling studies to determine the cell type specific complement of K-channel genes, and the ability to block specific channel genes through pharmacology or molecular methods may provide novel insights into how specific K-Channels help to determine the physiology of HVC.

In contrast with HVC, virtually every K-Channel gene that is exclusively differential in RA showed relatively low expression, including both K_(Na+)_ channel subunits (*KCNJ5* and *KCNJ6*), both KCNT channels, and a variety of KCNB and KCND accessory subunits. These results suggest an emphasis on stabilizing neuronal networks within RA, possibly related to the preservation the low song-to-song syllable variability in firing properties that has been observed in most RA neurons [72,73]. Interestingly, recordings from RA neurons in both anesthetized *in vivo* and *in vitro* slice preparations indicate that these neurons act like intrinsic pacemaker cells, often displaying extremely high rates of spontaneous activity [74]. This is in stark contrast with HVC neurons (all classes), which are relatively quiescent unless stimulated by current injection [30]. Thus, one explanation for the apparent decreased level of K-Channel mRNA expression in RA is the suppression of specific K-Channel variants (or their accessory subunits), depressing rapid changes in cellular excitability and firing adaptation.

## Conclusions

One of the largest and most structurally diverse families of ion-selective channels in vertebrates, K-Channels participate in a wide array of neuronal functions, ranging from resting membrane potential and intrinsic excitability to action potential repolarization and synaptic plasticity. Our genomics and *in situ* analyses have shed new light on a rich diversity of molecular components that are highly likely to shape biophysical and excitable properties of neurons in the songbird brain. Our findings indicate that K-Channels are expressed in various combinations in the song system, and point to specific genes that may play instrumental roles in the specialized physiological properties of individual song nuclei. This suite of K-Channels provides a battery of new targets for future research utilizing pharmacological and genetic techniques to manipulate brain nuclei, functional circuits, or even specific cell types. Understanding how these channels shape the properties of the song control circuitry will go a long ways towards improving our understanding of the neuronal requirements for vocal learning in songbirds and other vocal learners.

## Methods

### Searching for potassium (K-) channel genes in the zebra finch genome

To identify the complete set of conserved, novel, and expanded potassium channel and potassium channel-related genes (K-Channels) in the zebra finch genome we used a comprehensive strategy consisting of mRNA/protein alignments, syntenic analysis, and comparative genomics. First, we conducted a search of the HUGO Gene Nomenclature Committee website (HGNC; [32]) and retrieved a set of 123 unique genes in the human genome that have been annotated as K-Channel or K-Channel related. Three K-Channel pseudogenes (*KCNMB3P, KCTD9P1, KCTD9P2*) were included on this list, but would not be expected to influence cellular function. This list included all of the known members of the voltage-gated K-Channel family that have been recognized by the international union of basic and clinical pharmacology (IUPHAR; [33]). For each K-Channel gene we then used UCSC’s genomic Blast-like alignment tool (BLAT; [35][75,76]) to align corresponding human mRNA and predicted protein sequences (Ensembl GRCh37.p8, Feb 2009) against the zebra finch genome assembly (WUGSC 3.2.4/taeGut1; Jul 2008). This comprehensive approach served to identify any loci in zebra finch with sequence similarity to the initial human orthologs, including the actual orthologs as well as paralogs and allelic variants. This approach also served to identify loci with correctly and incorrectly annotated Ensembl gene model predictions, as well as novel loci not predicted by the current Genebuild (release 70) in zebra finches. To establish correct orthology, we conducted parallel BLAT searches by mapping the human sequences (mRNA and protein) onto the genomes of chicken (*Gallus gallus*), turkey (*Meleagris gallopavo*), lizard (green anole, *Anolis carolinensis*) or turtle (painted turtle, *Chrysemys picta* or Chinese softshell turtle, *Pelodiscus sinensis*), frog (African clawed frog, *Xenopus laevis*), fish (zebra fish, *Danio rerio*) and mouse (*Mus musculus*), and confirmed synteny by examining stretches of flanking genes. In some cases this approach helped to identify orthologs that were apparently absent or missing in other genomes. Finally, for each identified zebra finch ortholog, we re-BLAT-aligned its corresponding mRNA and predicted protein sequence against the zebra finch genome in order to identify additional loci corresponding to possible zebra finch specific gene duplications, gene expansions, or pseudogenes. In cases where we were unable to find direct evidence for K-Channel orthologs in the zebra finch genome assembly, we also used blastn (http://blast.ncbi.nlm.nih.gov) to search against available zebra finch EST/cDNA databases, and tblastn to search against translated peptide databases using query sequences derived from chicken, lizard, frog, fish, and other representative mammalian species to search for transcript evidence. Throughout, we refer to zebra finch genes, mRNAs and proteins using the HGNC nomenclature convention ([77]; uppercase italics for genes, and uppercase for protein).

### Searching for allelic variants

To identify possible allelic variants of K-Channel genes in zebra finch, we BLAT-aligned each ortholog and analyzed any BLAT alignments at a secondary locus that had an identity score > 95% sequence identity. Virtually all of these secondary alignments were to chromosome unknown (chrUn). We only considered alignments in regions of high quality sequence (i.e. regions with Phred quality scores > 50 for individual base calls), and ignored those with low BLAT alignment scores (e.g. < 95), or cases where the Ensembl model itself was derived from a region of poor sequence quality. Next, we aligned the mRNA sequence derived from the protein coding region for each Ensembl gene model to the mRNA sequence derived from the secondary genomic alignment (i.e. the allelic variant). All sequence analyses were performed using the eBioX (1.5.1) suite of analysis tools [78]. Specifically, pairwise alignments of protein and mRNA sequences were estimated using a Smith-Waterman local alignment algorithm [79]; multiple sequence alignments were conducted using the ClustalW alignment algorithm [80]. Individual nucleotide differences were then identified and categorized as synonymous (i.e. resulting in no change to the amino-acid sequence) or non-synonymous (i.e. resulting in a change to the amino-acid sequence). Non-synonymous changes were further characterized as being conservative (i.e. substitution resulted in an amino-acid whose side chains have similar biochemical properties), or non-conservative (i.e. a missense mutation resulting in the possible change in amino-acid sequence). Finally, for alleles showing non-conservative substitutions, we conducted a more thorough analysis of the predicted protein structure to determine whether amino changes were likely to affect known protein coding domains related to K-Channel function (e.g. transmembrane domain, pore-forming domain, amino/carboxy terminus).

### Searching for K-Channel genes with unique features in zebra finch versus chicken

We used comparisons of protein coding domains in chicken and zebra finch in order to identify orthologous K-Channel genes with high ratios of non-synonymous substitutions (dN) to synonymous substitutions (dS). High dN/dS ratio can be used as a positive indicator of selective pressure acting on a protein-coding gene. To conduct this analysis we first compiled dN/dS ratios for all members of the K-Channel gene family in the zebra finch in comparison with chicken. In cases where correct 1-to-1 orthologs have been identified by Ensembl, we retrieved the dN/dS ratios from Ensembl Compara [81]. In cases where the correct orthologs are present, but have not been predicted by Ensembl in the zebra finch, we first identified the orthologous predicted coding sequences based on the results of BLAT-alignments. In cases where the correct orthologs were split into more than one Ensembl model, or lacked specific domains compared to chicken due to incomplete model predictions, we reconstructed the complete predicted coding sequence by combining any partial models, or used multiple partial segments of the predicted sequence for our analysis. We note that in six cases we found that genes with particularly high dN/dS ratios as reported by Ensembl Compara were incorrect due to improperly aligned protein coding models, incomplete model predictions, and/or models derived from low quality sequence (Table S5A). We therefore verified, and using PAL2NAL [82] recalculated dN/dS ratio values for each gene based on segments of high quality sequence and visual confirmation of correct protein alignments (Table 1). We excluded all protein coding regions derived from low sequence quality and/or regions (and in some cases single exons) clearly missing in one of the species due to a gap in the current genomic sequence. In most cases these regional differences were not supported by EST evidence and likely represent artifacts resulting from automated gene model prediction algorithms. We then calculated the average dN/dS value for the entire K-Channel gene family (Table 1), and identified a subset of genes with particularly high values when compared to the group as a whole (i.e. > ~2× above the average value). Next, using Ensembl Protein Summary or, in cases were no Ensembl models were predicted, NCBI’s Conserved Domain Search [83], we determined whether any genes with high dN/dS ratios had non-conservative amino-acid substitutions occurring within protein coding domains related to channel function (e.g. ion transport domains, transmembrane domains, etc.). For such genes we also calculated dN/dS ratios for finch vs. lizard, and chicken vs. lizard ortholog pairs in order to establish whether these genes might also be evolving more rapidly in finch, chicken, or birds in general.

Next, we conducted a search for specific insertions/deletions in the protein coding sequences of the chicken and zebra finch orthologs. Specifically, we retrieved the alignments of the orthologous protein coding pairs of zebra finch and chicken from Ensembl Compara, and looked for segments that are absent in one or the other species. In several cases we first needed to reconstruct the correct predicted protein sequence in the zebra finch or chicken, as discussed above. We note that Ensembl model predictions are currently available only for the 2006 chicken assembly (Gallus_gallus-2.1). Therefore, whenever an insertion/deletion was identified, we confirmed its occurrence by examining the latest chicken genome assembly (Gallus_gallus-4.0). We note that by performing this additional curation step, we were able to determine that in several cases the apparent insertion/deletion in the previous assembly was in fact an artifact due to incompleteness or incorrectness of the Ensembl model prediction because of genomic gaps and/or low quality sequence. In cases where insertion/deletions were identified that are clearly not due to genomic gaps in either species, we next examined whether they occur within a known protein domain whose disruption might have functional implications. We also examined the insertion/deletion region in non-avian orthologs. In cases where the sequence in zebra finch resembled closely those in non-avian orthologs, we concluded that the insertion/deletion is likely chicken-specific and did not pursue further analyses. In cases where the sequence in chicken resembled closely those in non-avian orthologs, we asked whether similar insertion/deletions are also present in humans, to establish whether they might be characteristic of vocal learning species.

### Animal preparation

We used a total of 31 adult male zebra finches (*Taeniopygia guttata*) that were obtained from our own breeding colony or purchased from local breeders. Since this study was designed to primarily analyze the distribution of K-Channel genes in the zebra finch song system, and not their regulation by singing behavior, hearing, or other behavioral state, all birds were first isolated overnight (12:12-hour light–dark cycle) in custom-built acoustic isolation chambers to reduce non-specific auditory stimulation. On the following morning (~9:00 AM), birds were monitored for at least 1 hour, confirmed to be non-singing, and then sacrificed by decapitation. Brains were quickly removed, frozen, sectioned on a cryostat (10 μm) and prepared for *in situ* hybridization as described in detail in [84]. Animal protocols were approved by OHSU’s IACUC committee and are in accordance with NIH guidelines.

### cDNA selection and riboprobe synthesis

All probes were derived from cDNA clones selected from the ESTIMA zebra finch brain cDNA collection [38]. For each gene we selected a single cDNA clone based on a careful search against zebra finch ESTs and mRNAs that were partially overlapping or contiguous with the gene model based on a series of EST reads. However, in rare cases we also selected clones that were found to align within ~500 bp of the 3′-end of a gene model. Given the close proximity of these reads to the gene model, and in most cases evidence of polyadenylation (i.e. poly-A tail), we are reasonably confident that these cDNAs correspond to the 3′-end of the gene, and thus are suitable for use as *in situ* probes. Where possible we specifically selected clones containing long stretches of primarily non-coding or 3′-untranslated (3-UTR) EST sequence, since these regions typically are unlikely to contain conserved domains present in related members of the K-Channel gene family. To confirm this, we performed BLAT realignment of these ESTs to the zebra finch genome to confirm that they mapped to a single locus. We performed a similar analysis for ESTs containing known or predicted protein coding sequence and only selected clones that could be aligned to a single unambiguous locus.

DIG-labeled riboprobes were prepared as previously described ([84]; see also [85,86]). Briefly, we first isolated plasmid DNA from the Songbird ESTIMA cDNA clone collection, digested out the cDNA insert with a BSSHII restriction enzyme (New England Biolabs; Ipswich, MA), and twice purified the template with a GeneJet (Fermentas, NJ) or Qiagen PCR purification kit (Qiagen Inc., Valencia, CA). Antisense strand probes were synthesized at 37°C for 4–5 hours using T3 RNA polymerase (Promega Inc., Madison, WI) and DIG-labeling mix (Roche), and were purified by Sephadex G-50 columns.

### *In situ* hybridization

Methods for performing *in situ* hybridization on zebra finch parasagittal brain sections are detailed in [84] see also [85,86]). For this study, each probe was hybridized to a pair of brain sections containing major song control nuclei, namely HVC, RA, LMAN, and Area X. The hybridized sections allowed us to examine expression in a wide range of brain areas, nuclei and subdivisions. In cases where we observed marked evidence of regional differential expression, we extended the analysis further to a series of parasagittal sections; the resulting images will be included as part of a zebra finch molecular atlas of brain gene expression we are currently developing (ZEBrA; [87]). Using antisense probes derived from clones representing specific transcripts, we hybridized sections from at least three different brains to confirm the pattern and distribution of labeled cells. Briefly, brain sections were post-fixed in a 3% buffered paraformaldehyde solution for 5 min at room temperature (RT), rinsed twice in 0.1 M PBS, and dehydrated through an alcohol series. Sections were then acetylated for 10 min in a solution of 1.35% triethanolamine and 0.25% acetic anhydride in water, and rinsed three times with 2x SSPE containing in mM: 300 NaCl, 20 NaH_2_PO_4_-H_2_O, and 2.5 EDTA (pH 7.4). Each section was then hybridized with a solution (16 μl) containing 50% deionized formamide, 2x SSPE, 1 μg/μl tRNA, 1 μg/μl BSA, 1 μg/μl poly-A in DEPC-treated water, and 1 μl of DIG-labeled riboprobe. Slides were coverslipped, sealed by immersion in mineral oil, and incubated overnight at between 63-67°C, reflecting the optimal in situ conditions for each probe. The following day sections were rinsed in chloroform, de-coverslipped in 2x SSPE, and washed by incubating serially for 1 hr at RT in 2x SSPE, 1 hr at 63-67°C in 2× SSPE containing 50% formamide, and twice in 0.1x SSPE for 30 min at 63-67°C.

Sections were then blocked for 30 min at RT in TNB buffer (0.5% w/v of TSA-Biotin kit’s blocking reagent; 100 mM Tris; 150 mM NaCl; 1% skim milk; pH 7.5), and incubated for 2 hr in TNB with an alkaline phosphatase conjugated anti-DIG antibody (anti-DIG-AP; 1:300 dil., Roche Applied Science, Mannheim, Germany), washed 3 times for 5 min in TMN (100 mM Tris, 150 mM NaCl, 0.05 M MgCl; pH 9.5), and incubated for 1–3 days in a ready-to-use Tris-buffered solution containing the alkaline phosphatase substrates Nitro-Blue Tetrazolium Chloride (NBT; 0.42 g/L) and 5-Bromo-4-Chloro-3-Indolyl-phosphate p-Toluidine Salt (BCIP/NBT; 0.21 g/L; Substrate Solution NEL937, Perkin-Elmer, Waltham, MA). Slides were washed 3 times for 5 min at RT in TNT (TN with 0.3% Triton X-100), rinsed briefly in distilled water to remove salts, and coverslipped with Vectamount AQ (Vector Labs, Burlingame, CA). Slides were imaged at 0.42 μm/pixel (20×) using an Olympus Nanozoomer HT2. The resulting 8-bit color images were then converted to gray-scale, cropped, and contrast/brightness enhanced as needed in Photoshop CS5 (Adobe Systems Inc., San Jose, CA). Additional gray-scale balancing was also performed across images to ensure that background levels were similar across brain sections. Figures were prepared in Illustrator CS5 (Adobe Systems Inc.).

### Song system expression and marker analysis

We conducted a qualitative assessment of relative mRNA expression within the four telencephalic nuclei of the song system (RA, HVC, Area X, and LMAN) using a scoring system based on a scale of o (no detectable expression), and + to +++ (for low to high expression). Genes that were found to be differentially expressed in a song nucleus relative to surrounding brain tissue were further scored on a subjective scale consisting of either one or two up- or down-arrows indicating the approximate level of enrichment (or impoverishment) in song nuclei. This analysis was conducted through a comparison of expression over LMAN versus anterior Nidopallium, RA versus Arcopallium, HVC versus dorsocaudal Nidopallium (equivalent to the shelf region; [85,88]), and Area X versus medial Striatum. We note that we systematically perform controls where we omit probe or primary anti-dig antibody from the protocol, or for selected probes perform hybridization with sense strand riboprobes; none of these controls yield detectable cellular signal, further supporting the specificity of the hybridization results obtained.

## Abbreviations

A: Arcopallium; AFP: Anterior forebrain pathway; BLAT: Blast-like alignment tool; Cb: Cerebellum; CMM: Caudomedial mesopallium; CO: Optic chiasm; DLM: Medial part of the dorsolateral nucleus of the anterior thalamus; DMP: Direct motor pathway; Gcl: Granular cell layer of the cerebellum; GIRK: G-protein coupled inward rectifying K-Channel; GP: Globus pallidus; H: Hyperpallium; HVC: HVC proper name; lHb: Lateral habenula; K(Na+): Na^+^-sensitive K-Channels; LMAN: Lateral magnocellular nucleus of the anterior nidopallium; M: Mesopallium; Mcl: Molecular layer of the cerebellum; mHb: Medial habenula; N3: Oculomotor nerve; N: Nidopallium; NCM: Caudomedial nidopallium; nXIIts: Tracheosyringeal portion of the nucleus of the hypoglossal nerve (XII); OM: Occipitomesencephalic tract; OMt: Tract of the oculomotor nerve; Ov: Nucleus Ovoidalis; PAm: Nucleus para-ambiguus; Pcl: Purkinje cell layer of the cerebellum; RA: Robust nucleus of the arcopallium; RAm: Nucleus retroambiguus; St: Striatum; TeO: Optic tectum; V: Ventricle; VTA: Ventral tegmental area; X: Striatal area X.

## Competing interest

The authors declare that they have no competing interests.

## Authors’ contributions

Conceived and designed the experiments: PVL and CVM. Performed the experiments: JBC and PVL. Analyzed the data and wrote the paper: PVL and CMV. All of the authors have read and approved the final manuscript.

## Supplementary Material

Additional file 1 **An Excel file containing 5 separate sheets (Table S1-S5) that document specific Zebra finch K-Channel: (Table S1) Gene duplications, (Table S2) Gene deletions, (Table S3) Genes with alleles, (Table S4) Proteins with amino acid insertions/deletions, and (Table S5) Genes with high dN/dS values.** Click here for file

Additional file 2 **Alignment of amino acid sequences predicted for zebra finch *KCNJ3 *(ENSTGUG00000012153) and *KCNJ3L *(ENSTGUG00000002970).** Numbers on the left indicate the relative position of amino acid residues in each sequence; identical residues are shaded in black. Notably, the 5′-end of KCNJ3 appears to be missing due to gap in the genomic sequence. Highly conserved motifs that define the signature for KCNJ sub-family members are indicated in blue, those specific to the KCNJ3 subunit are indicated in red. Click here for file

Additional file 3 **Alignment of amino acid (AA) residues predicted from *KCNJ5/9 L *orthologs in zebra finch, chicken, and lizard.** The numbers on the left indicate the relative position of residues in each sequence; residues shaded in black are identical, those in gray indicate a conservative substitution. Notably, the chicken copy has a large 145 AA insert at residue 22, while zebra finch has a smaller 30 AA insert at position 291. The positions of highly conserved motifs (1–6) that define the KCNJ sub-family of K-Channels are indicated in blue. Click here for file

Additional file 4 **Alignment of amino acid (AA) sequences predicted from *KCNK16L *orthologs in lizard, frog, zebra finch, chicken, platypus, and horse.** The numbers on the left indicate the relative position of AA residues in each sequence; AA residues shaded in black are identical, those in gray indicate a conservative substitution. The positions of highly conserved TASK and KCNK channel sequence motifs are indicated in blue and red, respectively. Click here for file

Additional file 5 **Alignment of amino acid (AA) sequences predicted from *KCNV2L *orthologs in zebra finch, chicken, and frog.** The numbers on the left indicate the relative position of AA residues in each sequence; AA residues are shaded in black when they are identical in >50% of species, and grey when the substitution is conservative. The positions of conserved KCN (voltage-gated) family sequence motifs are indicated in red; conserved sequence motifs that define the KCNV sub-family of channels are indicated in green. Click here for file

Additional file 6 **Alignment of amino acid (AA) sequences predicted from KCNQ1L orthologs in zebra finch, chicken, and frog.** The numbers on the left indicate the relative position of AA residues in each sequence; AA residues are shaded in black when they are identical in > 50% of species, and grey when the substitution is conservative. The positions of several of the nine conserved KCNQ Channel sequence motifs are indicated in red. Click here for file
