# Supplementary material for: Genomics analysis of potassium channel genes in songbirds reveals molecular specializations of brain circuits for the maintenance and production of learned vocalizations
Source: BMC Genomics. 2013 Jul 11;14:470. doi: 10.1186/1471-2164-14-470 (PMC3711925; doi:10.1186/1471-2164-14-470)
Supplement: Additional file 1 — An Excel file containing 5 separate sheets (Table S1-S5) that document specific Zebra finch K-Channel: (Table S1) Gene duplications, (Table S2) Gene deletions, (Table S3) Genes with alleles, (Table S4) Proteins with amino acid insertions/deletions, and (Table S5) Genes with high dN/dS values. [file 1471-2164-14-470-S1.docx]

| ***Table S1. True gene duplications and erroneously identified duplications in the zebra finch genome.*** | | | |  |
| --- | --- | --- | --- | --- |
|  |  |  |  |  |
| **Revised Ensembl Annotation** | **Current Ensembl Annotation (taegut3.2.4 )** | **Ensembl Model or Chromosome location** | **Conclusions/ Implications** |  |
| KCNE1 | KCNE1 | ENSTGUG00000016311 | Locus Chr1B_random:70,101-70,314 (KCNE1P) is an apparent duplication of KCNE1 that is unique to zebra finch (Fig. 2C). |  |
| KCNE1P | Not Predicted | Chr1B_rand:70101-70314 |  |  |
| KCNJ3 | Uncharac. Protein | ENSTGUG00000012153 | ENSTGUG00000002970 (KCNJ3L) is an apparent duplication of KCNJ3 that occurred in euteleostomi, but was lost in mammals (Figs. 2A/B, S1). |  |
| KCNJ3L | KCNJ3 | ENSTGUG00000002970 |  |  |
| KCNJ5 | KCNJ5 | ENSTGUG00000000586 | KCNJ5/9L is an apparent duplication of KCNJ5 or KCNJ9 that occurred in euteleostomi, but was possibly lost in mammals (Figs. 2D, S2). |  |
| KCNJ9 | KCNJ9 | ENSTGUG00000015501 |  |  |
| KCNJ5/9L | Not predicted | Chr24:252787-253805 |  |  |
| KCNK16 | Not predicted | Chr3:29820700-29833238 | Locus (Chr3:29,820,700-29,833,238) is the ortholog of KCNK16. ENSTGUG00000013021 (KCNK16L) is an apparent duplication of KCNK16 that is conserved among tetrapods, and possibly lost in teleosts (Figs. 2E/F, S3). |  |
| KCNK16L | KCNK16 | ENSTGUG00000013021 |  |  |
| KCNV2 | KCNV2 | ENSTGUG00000005377 | ENSTGUG00000000100 (KCNV2L) is an apparent duplicaton of KCNV2 in tetrapods that was likely lost in some mammals (Figs. 3A/B, S4). |  |
| KCNV2L | XP_002189733.1 | ENSTGUG00000000100 |  |  |
| KCNQ1 | KCNQ1 | ENSTGUG00000009167 | ENSTGUG00000012696 (KCNQ1L) is an apparent duplication of KCNQ1 that occurred in tetrapods or euteleostomi, but was lost in mammals (Figs 3C/D, S5). |  |
| KCNQ1L | novel gene | ENSTGUG00000012696 |  |  |
| KCTD12 | KCTD12 | ENSTGUG00000012550 | ENSTGUG00000012550 is the true ortholog of KCTD12. ENSTGUG00000005525 (KCTD12L) is an apparent duplication of KCTD12 that occurred in euteleostomi, but was lost in humans. |  |
| KCTD12L | KCTD12 | ENSTGUG00000005525 |  |  |
|  |  |  |  |  |

***Table S2: Genes identified as missing (or deleted) in various vertebrate lineages.***

| **Vertebrate Lineages** | **Rev. HUGO term** | **Comments** |
| --- | --- | --- |
| **Missing in zebra finch (possibly in songbirds)** | **KCNC3** | Gene and syntenic group from humans (ENSG00000131398 on chr19:50,638,455-51,012,944) present in lizard ENSACAG00000005085 on scaffold_306:59,536-376,313), and chicken (LOC1008859454; chrUn_AADN03014657:49-889). |
|  | **KCNE1L** | Present in chicken (ENSGALG00000023613 on chr4:13,896,732-13,913,718). In finch, only flanking genes present (chr4A:12,167,461-12,197,015); no gaps in the region. |
|  | **KCNE2** | Present in chicken (IPI00580354.3 on chr1:109,290,593-109,310,514). In finch, only flanking genes present (chr1B_random:118,919-127,120); gene could be in a gap. |
|  | **KCNH2** | Gene and syntenic group from humans (ENSG00000055118 on chr7:150,510,185-150,806,878) present in frog (ENSXETG00000006509 on the small scaffold_1650:1-41,040 and syntenic group on scaffold_1193:1-83,367), absent in lizard (syntenic gene NOS3 present but next to a gap on scaffold_1388:1-98,310, so gene could be in the gap), and present in chicken (LOC100858122). |
|  | **KCNK3** | Gene and part of the synteny group from humans (ENSG00000171303 on chr2:26,772,984-27,094,193) present in lizard (ENSACAG00000006591 on scaffold_5187:1-7,216; the rest of the human synteny group not assembled in lizard), and in chicken (NC_006090.3). |
|  | **KCNU1** | Present in chicken (ENSGALG00000001560 on chr22:1,833,056-2,282,646). In finch, only flanking genes present (chr22:2,319,790-2,775,657); gene could be in a gap (a short model in this region seems unreloned as it has no hits to chicken upon blon or blast searches). |
|  | **KCTD19** | Present in chicken (IPI00819237.1 on chr11:1,176,176-1,276,453). In finch, only flanking genes present (chr11:4,995,028-5,152,245); gene could be in a gap. |
|  | **KCNIP3** | Gene and the syntenic group from humans (ENSG00000115041 on Chromosome 2: 95,963,052-96,051,825) present in Chinese softshell turtle (ENSPSIG00000008584 on JH210447.1:2170658-2182893:1). Human gene model aligns to chicken (chrUn_AADN03020325:1-1,697), but no sytneny is present. In finch, part of syntenic group is found on chr22:693,513-698,462 (no model) and the other on chrUn:60214694-60215948 (ENSTGUG00000013905). |
|  |  |  |
| **Missing in birds** | **KCNA7** | Gene and syntenic group from humans (ENSG00000104848 on chr19:49,570,675-49,576,198) present in lizard (ENSACAG00000013311 on scaffold_270:1,236,441-1,299,863) but absent in birds; one syntenic gene from humans present in finch on chromosome unknown. |
|  | **KCNAB3** | Gene and syntenic group from humans (ENSG00000170049 on chr17:7,727,222-7,931,557) present in lizard (ENSACAG00000004127 on scaffold_1129:1-181,742) but absent in birds. |
|  | **KCND1** | Gene and part of the syntenic group from humans (ENSG00000102057 on chrX:48,713,944-48,932,945) present in lizard (ENSACAG00000007541 and ENSACAG00000007542.1 on scaffold_32:3,552,720-3,960,595) but absent in birds. Part of the syntenic group from lizard present in birds, but flanking genes from lizard absent in birds. |
|  | **KCNH3** | Gene and syntenic group from humans (ENSG00000135519 on chr12:49,856,388-50,028,629) present in lizard (ENSACAG00000002637 on scaffold_1166:1-188,065, with a possible gene duplication - ENSACAG00000010414 on scaffold_174:1,865,759-2,059,356) but gene absent in birds. Flanking genes from humans present in chicken (chrE22C19W28_E50C23:850,702-869,426) and one flanking gene present in finch (chrUn:162,062,761-162,110,388). |
|  | **KCNJ14** | Gene and syntenic group from humans (ENSG00000182324 on chr19:48,916,358-49,011,775) present in lizard (ENSACAG00000016771 on scaffold_76:2,309,938-2,624,865) but absent in birds. |
|  | **KCNK6** | Gene and syntenic group from humans (ENSG00000099337 on chr19:38,773,965-38,869,079) present in lizard (ENSACAG00000003911 on scaffold_53:2,513,812-2,589,816) but absent in birds. |
|  | **KCNN4** | Gene and syntenic group from humans (ENSG00000104783 on chr19:43,926,854-44,523,220) present in lizard (ENSACAG00000004522 on scaffold_53:4,428,566-4,584,517) but absent in birds. |
|  | **KCTD13** | Gene and syntenic group from humans (ENSG00000174943 on chr16:29,859,700-29,995,301) present in lizard (ENSACAG00000014374 on scaffold_76:6,697-171,315) but absent in birds. |
|  |  |  |
| **Missing in birds and lizards** | **KCNK4** | Gene and syntenic group from humans (ENSG00000182450 on chr11:64,019,045-64,107,250) present in frog (ENSXETG00000007210 on scaffold_585:390,374-698,693) but absent in birds and lizard. |
|  | **KCNMB3** | Gene and syntenic group from humans (ENSG00000171121 on chr3:178,848,329-179,094,046) present in frog (ENSXETG00000007254 on scaffold_3:7,069,431-7,175,531) but gene absent in birds and lizard. Syntenic group from humans present in finch (chr9:20,100,609-20,184,007) and chicken (chr9:18,744,942-18,830,201) but absent in lizard. |
|  |  |  |
| **Missing in birds, lizard, frog and fish (possibly unique to mammals)** | **KCNK7** | Gene and syntenic group in homo (ENSG00000173338 on chr11:65,330,084-65,393,709) absent in fish (some orthologs in different species annotated as KCNK7 but located in different syntenic regions and more likely independent duplications from different paralogues), frog (some syntenic genes - EHBP1L1, SSSCA1 and RELA - present on different short scaffolds), lizard (one syntenic gene - MAP3K11 - present on short scaffold_1794:1-39,918) and birds. Possibly unique to mammals. |
|  | **KCTD11** | Gene and syntenic group from humans (ENSG00000213859 on chr17:7,198,344-7,322,111) absent in fish (ENSGACG00000015290 on scaffold_48: 831,935-834,578 of stickeback annotated as KCTD11 but located in a different syntenic region and more likely an independent duplication from a different paralogue), frog (one human syntenic gene - acap1 - present - ENSXETG00000017847 on scaffold_1512: 16,680-51,739), lizard and birds. Possibly unique to mammals. |

***Table S3. Zebra finch genes that possess alleles with synonymous and non-synonymous substitutions***

| **HUGO** | **Main Model** | **Allele Model/ Location** | **Subst.^%^** | **Comments** |
| --- | --- | --- | --- | --- |
| KCNH7 | ENSTGUG00000006950 | Non-overlapping segments at chr7:11,991,762-11,991,872; chrUn_67,799,166-67,799,666; chrUn_57,328,343-57,328,599. | NC | D for N (SQTT**D**DTDG / SQTT**N**DTDG) at residue 44 that could change/modulate PAS signal domain function. |
| KCNK10 | ENSTGUG00000012403 | chr5:43,841,058-43,841,388 | NC | A for T (PQFS**A**SSRP / PQFS**T**SSRP) residue 882 within a region with no predicted functional domains. |
| KCNJ8 | ENSTGUG00000012087 | ENSTGUG00000016073 (chrUn:19011335-19011900) | S (several) | Not expected to have functional consequences |
| KCNK16L | ENSTGUG00000013021 | Hiqh qualtiy non-overlapping segments at chrUn:61,648,590-61,655,793; chrUn:61,652,391-61,652,511 | S; NC (4) | (A) S for Y (QNGV**S**PVGN / QNGV**Y**PVGN) at residue 78; (B) single codon deletion of F: SNSF**F**FAGT/SNSF**_**FAGT at residue 98. The latter substitution could change/modulate ion transport function. |
| KCNH8 | ENSTGUG00000003211 | Non-overlapping segments at chrUn:4,142,607-4,142,856; chrUn:50,989,424-50,989,634 | S; C | (A) single codon insertion of S at residue 83 that could change/modulate PAS signal domain function; (B) S for T (NNPL**S**WDIG/NNPL**T**WDIG) at residue 366 that could change/modulate ion transport function. |
| KCNJ6 | ENSTGUG00000004993 | chrUn:131,626,640-131,627,039 | S (4) | Not expected to have functional consequences |
| KCNQ1 | ENSTGUG00000009167 | chrUn:10,948,461-10,948,583 | S | Not expected to have functional consequences |
| KCNQ2 | ENSTGUG00000007434 | chrUn:129,172,958-129,174,028 | S (several) | Not expected to have functional consequences |
| KCNK18 | ENSTGUG00000011054 | chrUn:76,065,026-76,067,538 | S (2) | Not expected to have functional consequences |
| KCTD4 | ENSTGUG00000018368 | ENSTGUG00000018204 | S | Not expected to have functional consequences |
| KCTD7 | ENSTGUG00000005372 | chr19:6,287,122-6,295,480 | S | Not expected to have functional consequences |
| KCTD9 | ENSTGUG00000004348 | chr22:1731380-1733407 | S (several) | Not expected to have functional consequences |
| % Subst. (Amino Acid Substitutions): S=Synonymous; C = Conservative; NC = Nonconservative | | | | |

| ***Table S4. Zebra finch predicted K-channel proteins with amino acid insertions/deletions (indels) as compared to chicken orthologs.*** | | | | | | |
| --- | --- | --- | --- | --- | --- | --- |
|  |  |  |  |  |  |  |
| **Revised HUGO term** | **Ensembl Models Analyzed** | **Indel Differences** | **Indel Conclusions** | **Indel Implications** | **Indel conservation (mammals)** | **Implications** |
| KCNA4 | ENSTGUG00000004781; ENSGALG00000012142 | NG missing at residue 681 | Real deletion within ion transport domain. | Possible change in ion transport kinetics. | NG plus other residues present in mouse. | Unclear. Region variable across taxa. |
| KCNA6 | ENSTGUG00000011959; ENSGALG00000017281 | (1) D missing at residue 80; (2) PL insertion at residue 216; (3) KQ inserted at residue 300. | Real insertions and a deletion within tetramerization domain (1) and an ion transport domain (2,3). | Possible change in channel tetramerization and/or ion transport kinetics. | All three regions are different between birds and mice. | Unclear. Region variable across taxa. |
| KCNAB2 | ENSTGUG00000000265; ENSGALG000000009128 | N inserted at residue 111; G missing at residue 126 | Analysis inconclusive. Insertion and deletion occur at the edge of differentialy spliced exon based on mRNA evidence in finch. | Functional consequence of splice variants unclear. | Not examined | Not examined |
| KCNB2 | ENSTGUG00000011511; ENSGALG00000022800 | LH missing at residue 501 | Real deletion within a region with no domain predictions. | Not predicted to affect channel function. | Region different between birds and mice. | Unclear. Region variable across taxa. |
| KCNG3 | ENSTGUG00000003439; ENSGALG00000009919 | GKRRA insertion at residue 139 | Real insertion within a region with no domain predictions. | Not predicted to affect channel function. | Mouse like zebra finch. | Chicken specific indel |
| KCNC4 | ENSTGUG00000000883; ENSGALG00000000400 | L missing at residue 147 | Real deletion within a region with no domain predictions. | Not predicted to affect channel function. | Mouse like zebra finch. | Chicken specific indel |
| KCND3 | ENSTGUG00000013643; ENSGALG00000001512 | (1) R missing at residue 429; (2) F inserted at residue 436; (3) T missing at residue 457. | Real insertion and deletions within regions with no domain predictions. | Not predicted to affect channel function. | Mouse and human orthologs like chicken | Finch specific indel |
| KCNIP2 | ENSTGUG00000009911; ENSGALG00000007666 | TVL insertion at residue 37. | Possibly real insertion at edge of splice junction within a region with no domain predictions. | Not predicted to affect channel function. | Mouse and Human like chicken. | Finch specific indel |
| KCNH1 | ENSTGUG00000003202; ENSGALG00000009877 | VIAKYGHLILVIFCSCSCCLLFGMENE inserted at residue 291. | Possibly real insertion within region with no predicted domains. Difference corresponds to single exon that not supported by EST or RefSeq support. | Not predicted to affect channel function. | Mouse like zebra finch. | Chicken specific indel |
| KCNT2 | ENSTGUG00000004186; ENSGALG00000002451 | Exon insertions and deletions. | Exons appear to be species specific. There are no ESTs or Refseqs to confirm exon predictions. | Functional consequence of splice variants unclear. | Not examined | Not examined |
| KCNJ5/9L | Chr24:252787-253805; ENSGALG00000006922 | GNGPRAMQGW KPLARQQGLL VARLSHCLAA inserted at residue 290. | Possible real insertion at the edge of splice junction within a region that contains a KNCJ family protein motif. No ESTs available to corroborate gene structure. | Functional consequences unclear. | Gene not present in mammals. | Gene not present in mammals. |
| KCNJ16 | ENSTGUG00000015743; ENSGALG00000004373 | PFS insertion at residue 343 | Real insertion within a region with no domain predictions. | Not predicted to affect channel function. | Mouse and human like chicken. | Finch specific indel |
| KCNK5 | ENSTGUG00000007722; ENSGALG00000010065 | NM missing at residue 325. | Real deletion within a region with no domain predictions. | Not predicted to affect channel function. | Not examined | Not examined |
| KCNK13 | ENSTGUG00000012450; ENSGALG00000010672 | SN insertion at residue 369 | Real insertion within a region with no domain predictions. | Not predicted to affect channel function. | Variable AA's present in mouse and human at this position | Chicken specific indel |
| HCN1 | ENSTGUG00000002345; ENSGALG00000014875 | SS missing at residue 511. | Real deletion within a region with no domain predictions. | Not predicted to affect channel function. | Variable AA's present in mouse and human at this position | Finch specific indel |
| KCTD12L | ENSTGUG00000005525/ FE734190; ENSGALG00000009628 | SAGAGGAGGT missing at residue 175 of FE734190 | Real deletion supported by EST evidence. Deletion is likely smaller than suggested by Ensembl. | Not predicted to affect channel function. | Not examined | Not examined |
| KCTD20 | ENSTGUG00000000921; ENSGALG00000000511 | DWL at residue 10; A missing at residue 45; T missing at residue 59 | Real deletions within a region with no domain predictions. | Not predicted to affect channel function. | Not examined | Not examined |

| ***Table S5. Zebra finch predicted K-channel proteins with high dN/dS ratio values as compared to chicken orthologs.*** | | | | | | |
| --- | --- | --- | --- | --- | --- | --- |
|  |  |  |  |  |  |  |
| **Revised HUGO term** | **Ensembl Model or Chrom. Location** | **Chicken Ortholog Ensembl Gene ID*** | **Ensembl dN/dS Ratio vs. Chicken** | **Revised dN/dS** | **dN/dS Comment** | **High dN/dS Implication** |
| **A - Corrected dN/dS Values** | |  |  |  |  |  |
| KCNAB1 | ENSTGUG00000011240; ENSTGUG00000011253 | ENSGALG00000010269 | 0.13; 0.02 | 0.02 | Ensembl dN/dS value incorrect due to low quality sequence, gaps, and assembly artifacts in zebra finch. dN/dS value recalculated using segments of high quality sequence from improved finch model prediction and chicken RefSeq NM_204906. |  |
| KCNC2 | ENSTGUG00000007354 | ENSGALG00000010204* | 0.25 | 0.01 | Ensembl dN/dS value incorrect due to low quality sequence, and assembly artifacts in zebra finch. Analysis partial for two segments in finch: 1-102 and 197-509. Prediction of segment 103-196 not supported by Refseq or EST. Segment 153 - 252 in chicken model is likely in a gap in finch |  |
| KCNC4 | ENSTGUG00000000883 | ENSGALG00000000400* | 0.17 | 0.03 | Ensembl dN/dS ratio incorrect due to low quality sequence, gaps, and assembly artifacts in zebra finch. Partial analysis conducted for two segments. Finch 1- 254; 311- 363, and chicken 1-443; 507-579. Finch segment 255 - 310 excluded due to low quality sequence. Chicken segment 444-506 excluded as it is likely in gap in finch. |  |
| KCNIP2 | ENSTGUG00000009911 | ENSGALG00000007666 | 0.14 | 0.03 | Ensembl dN/dS value incorrect due to low quality sequence in chicken. Correct dN/dS recalculated using high quality 2011 chicken assembly after removal of non-orthologous exons. |  |
| KCNN2 | ENSTGUG00000001344 | ENSGALG00000002539 | 0.36 | 0.04 | Ensembl dN/dS value incorrect due to inproper alignment of non-orthologous exons, and segments of low quality sequence. dN/dS ratio recalculated after removing the first exon from the finch model due to low quality sequence, and adding a 6th exon to the chicken model that is clearly present in the 2011 assembly. |  |
| KCTD3 | ENSTGUG00000002895 | ENSGALG00000009678 | 0.23 | 0.01 | Ensembl dN/dS value incorrect due to improper alignment of non-orthologous exons. dN/dS value was recalculated after removal of unsupported exons, improved model prediction, and protein realignment. Finch model residues 108-157 are likely false since they are not present in chicken, and are not supported by EST evidence. Chicken residue 130-169 of CHEST BU269561 are likely real, and are present in zebra finch. |  |
| **B - Confirmed High dN/dS Value Channels** | | |  |  |  |  |
| KCNG2 | ENSTGUG00000006664 | ENSGALG00000012652 | 0.11 | 0.11 | dN/dS value confirmed. High quality sequence and proper alignment of protein coding sequences. | Segments of divergence (~174-195; 470-500) contain no predicted domains. |
| KCNMB1 | ENSTGUG00000014898 | ENSGALG00000002118 | 0.14 | 0.14 | dN/dS value confirmed. High quality sequence and proper alignment of protein coding sequences. | 4 residue substitutions are present within a region that contains a predicted transmembrane domain (residues 160-175). No models in lizard or frog Turtle vs Chicken dN/dS = 0.08 (identity 89.3%), and turtle vs finch dN/dS = 0.17 (81.4%). |
| KCNK5 | ENSTGUG00000007722 | ENSGALG00000010065 | 0.14 | 0.14 | dN/dS value confirmed. High quality sequence and proper alignment of protein coding sequences. | Region of divergence is within the 3' half of the gene in a region that contains no predicted domains. |
| KCNK10 | ENSTGUG00000012403 | ENSGALG00000010598 | 0.23 | 0.23 | Recalculated dN/dS value after removing the 5' most exon in chicken and finch. First exons either correspond to alternate start sites, splice variants, or poor model predictions. | All substitutions occur within regions that contain no predicted domains. |
| KCNK16 | Chr3:29820700-29833238 | ENSGALG00000020049 | N/A | 0.18 | dN/dS value calculated by retrieving sequence for the corresponding locus in finch via genome alignment of the chicken model. The 5th exon was excluded from the analysis due to low quality finch sequence. The 1st predicted exon in chicken was also excluded since there is no support for this small exon. | Substitutions lie within regions that contain ion transport and transmembrane domains. Lizard vs chicken dN/dS = 0.22 (identity 79.2%); lizard vs finch dN/dS = 0.34 (71.3%). |
| KCNK16L | ENSTGUG00000013021 | ENSGALG00000012021* | 0.12 | 0.12 | dN/dS value confirmed. High quality sequence and proper alignment of protein coding sequences. | Segments of divergence occur in regions that contain (1-20) or are between (220-235) transmembrane domains, or are not predicted to contain a domain (65-90). No Ensembl model in lizard, but there is an EST. Partial alignment of lizard EST vs chicken dN/dS = 0.2 (identity 71.6%); lizard EST vs finch dN/dS = 0.19 (73.1%). |
| KCNK17 | ENSTGUG00000007716 | ENSGALG00000010068 | 0.24 | 0.24 | dN/dS value confirmed. High quality sequence and proper alignment of protein coding sequences. | The region of highest divergence occurs at the 5' end of the gene and contains no predicted domains. |
| KCNK18 | ENSTGUG00000011054 | ENSGALG00000009265 | 0.30 | 0.23 | Ensembl dN/dS value incorrect due to low quality sequence in zebra finch. Analysis partial for two segments in finch 1-180 and 290-393. Sequence between 181-289 is low quality, introducing an artificial frameshift. Sequence also not supported by allele location on Chr_Un:76065026-76067538. | The region of highest divergence occurs at the 5' end of the gene within a region that contains a transmembrane and the ion transport domain. Lizard vs chicken dN/dS = 0.16 (identity 60.5%); Lizard vs finch dN/dS = 0.13 (67.0%). |
| KCTD18 | Chr7:22311560-22315242 | ENSGALG00000008155 | N/A | 0.41 | dN/dS value calculated by first retrieving sequence from finch based on a partial alignment of ENSGAL000000008155. | Region of divergence is within the 3' half of the gene in a region that contains no predicted domains. |
| KCNRG | ENSTGUG00000012112 | ENSGALG00000017012 | 0.18 | 0.18 | dN/dS value confirmed. High quality sequence and proper alignment of protein coding sequences. | Several substitutions lie within a region that contains the tetramerization domain that mediates subunit interactions. Lizard vs chicken dN/dS = 0.26 (67.2%); Lizard vs finch dN/dS = 0.17 (68.1%). |
