# Supplementary figures and images for: Genomics analysis of potassium channel genes in songbirds reveals molecular specializations of brain circuits for the maintenance and production of learned vocalizations
Source: BMC Genomics. 2013 Jul 11;14:470. doi: 10.1186/1471-2164-14-470 (PMC3711925; doi:10.1186/1471-2164-14-470)

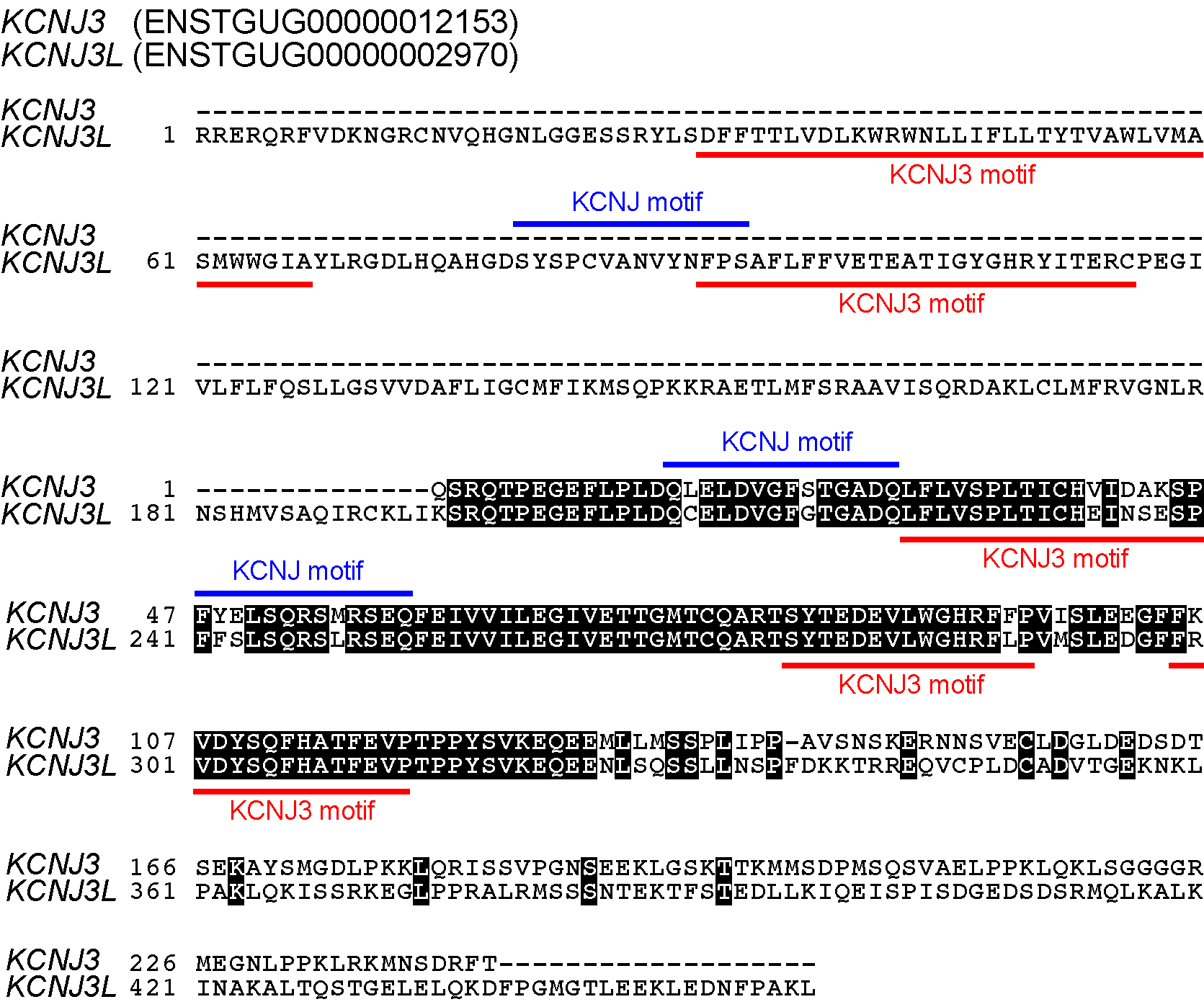

Supplement: Additional file 2 — Alignment of amino acid sequences predicted for zebra finch KCNJ3 (ENSTGUG00000012153) and KCNJ3L (ENSTGUG00000002970). Numbers on the left indicate the relative position of amino acid residues in each sequence; identical residues are shaded in black. Notably, the 5′-end of KCNJ3 appears to be missing due to gap in the genomic sequence. Highly conserved motifs that define the signature for KCNJ sub-family members are indicated in blue, those specific to the KCNJ3 subunit are indicated in red. [file 1471-2164-14-470-S2.tiff]

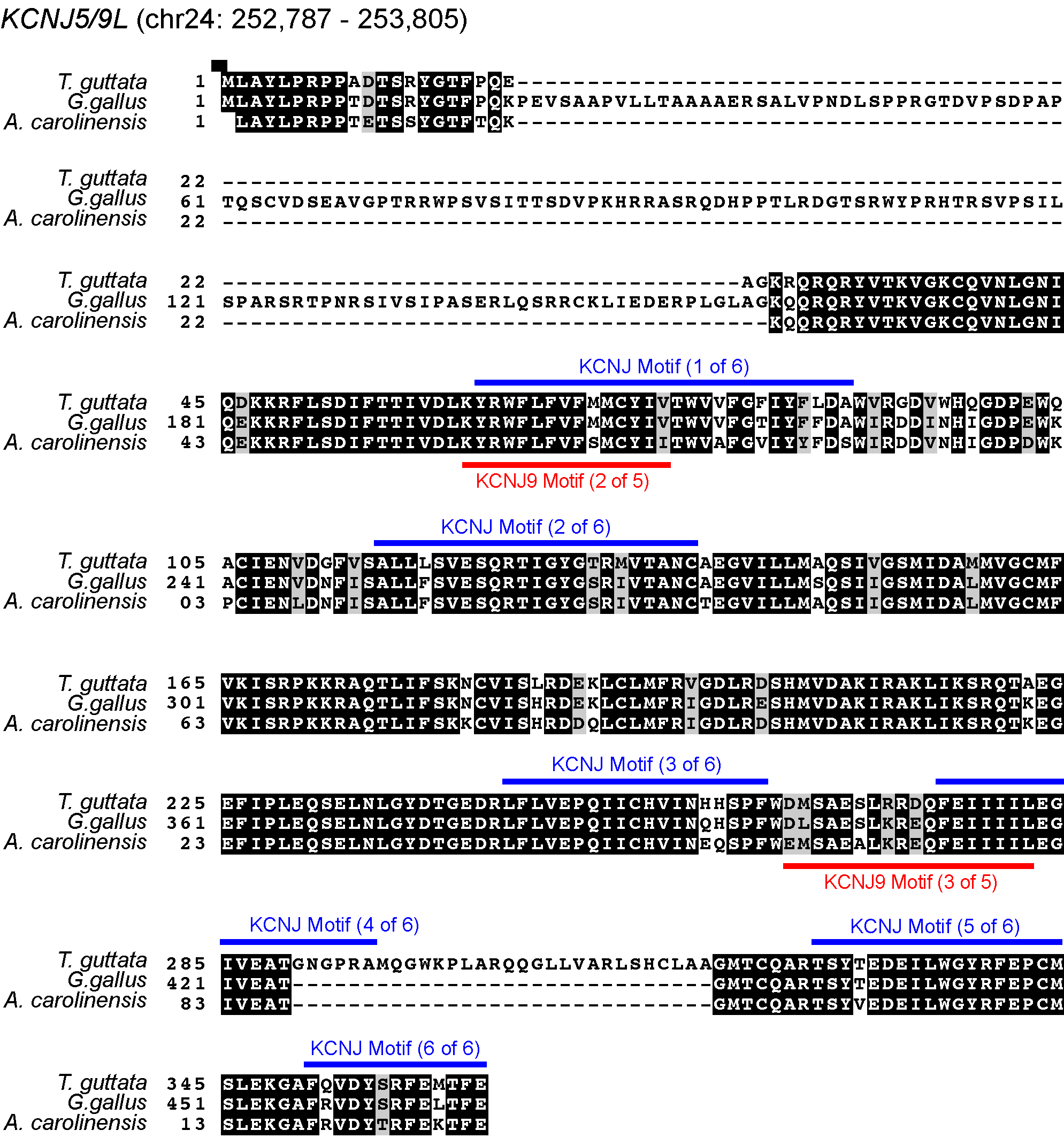

Supplement: Additional file 3 — Alignment of amino acid (AA) residues predicted from KCNJ5/9 L orthologs in zebra finch, chicken, and lizard. The numbers on the left indicate the relative position of residues in each sequence; residues shaded in black are identical, those in gray indicate a conservative substitution. Notably, the chicken copy has a large 145 AA insert at residue 22, while zebra finch has a smaller 30 AA insert at position 291. The positions of highly conserved motifs (1–6) that define the KCNJ sub-family of K-Channels are indicated in blue. [file 1471-2164-14-470-S3.tiff]

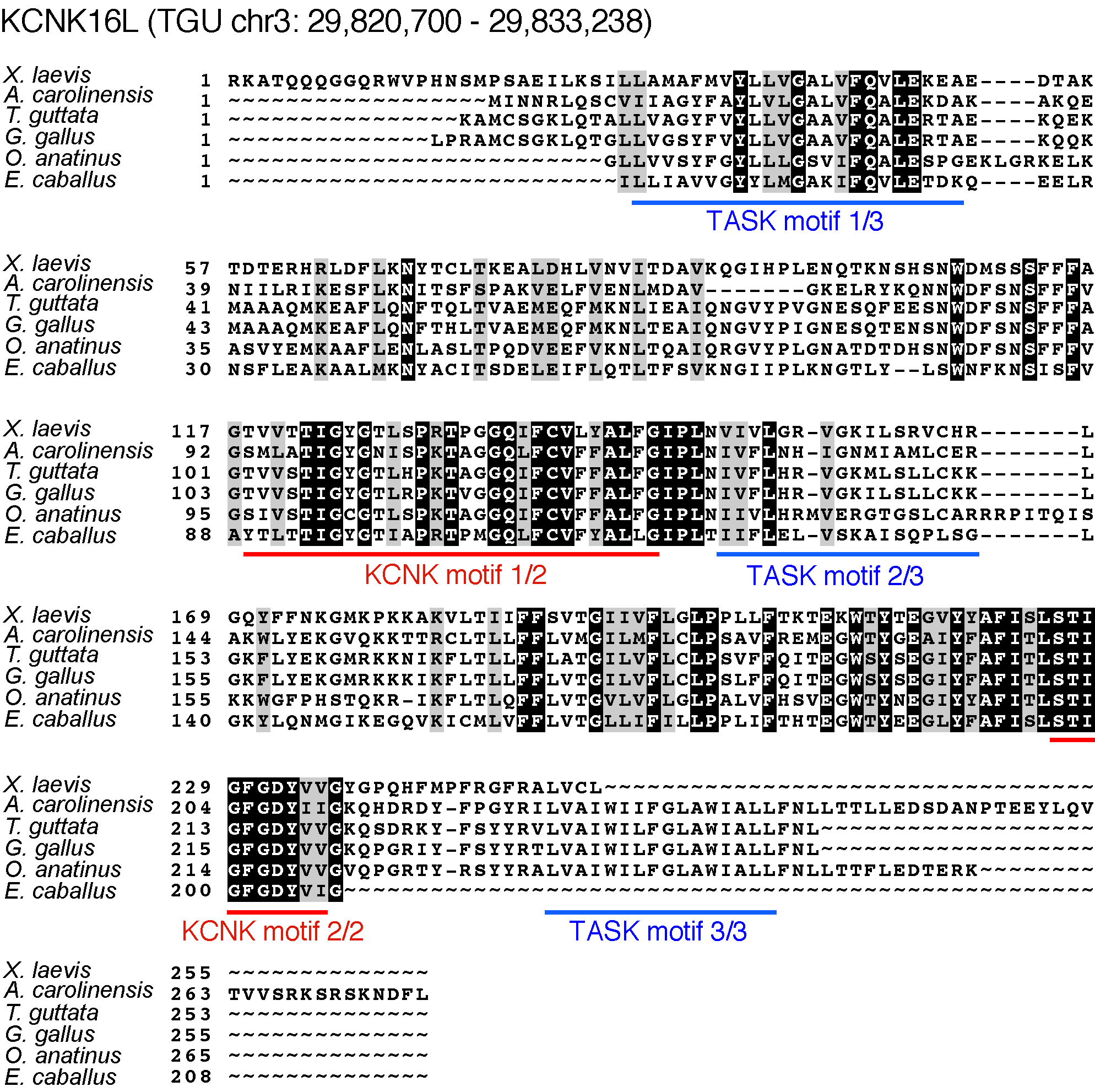

Supplement: Additional file 4 — Alignment of amino acid (AA) sequences predicted from KCNK16L orthologs in lizard, frog, zebra finch, chicken, platypus, and horse. The numbers on the left indicate the relative position of AA residues in each sequence; AA residues shaded in black are identical, those in gray indicate a conservative substitution. The positions of highly conserved TASK and KCNK channel sequence motifs are indicated in blue and red, respectively. [file 1471-2164-14-470-S4.tiff]

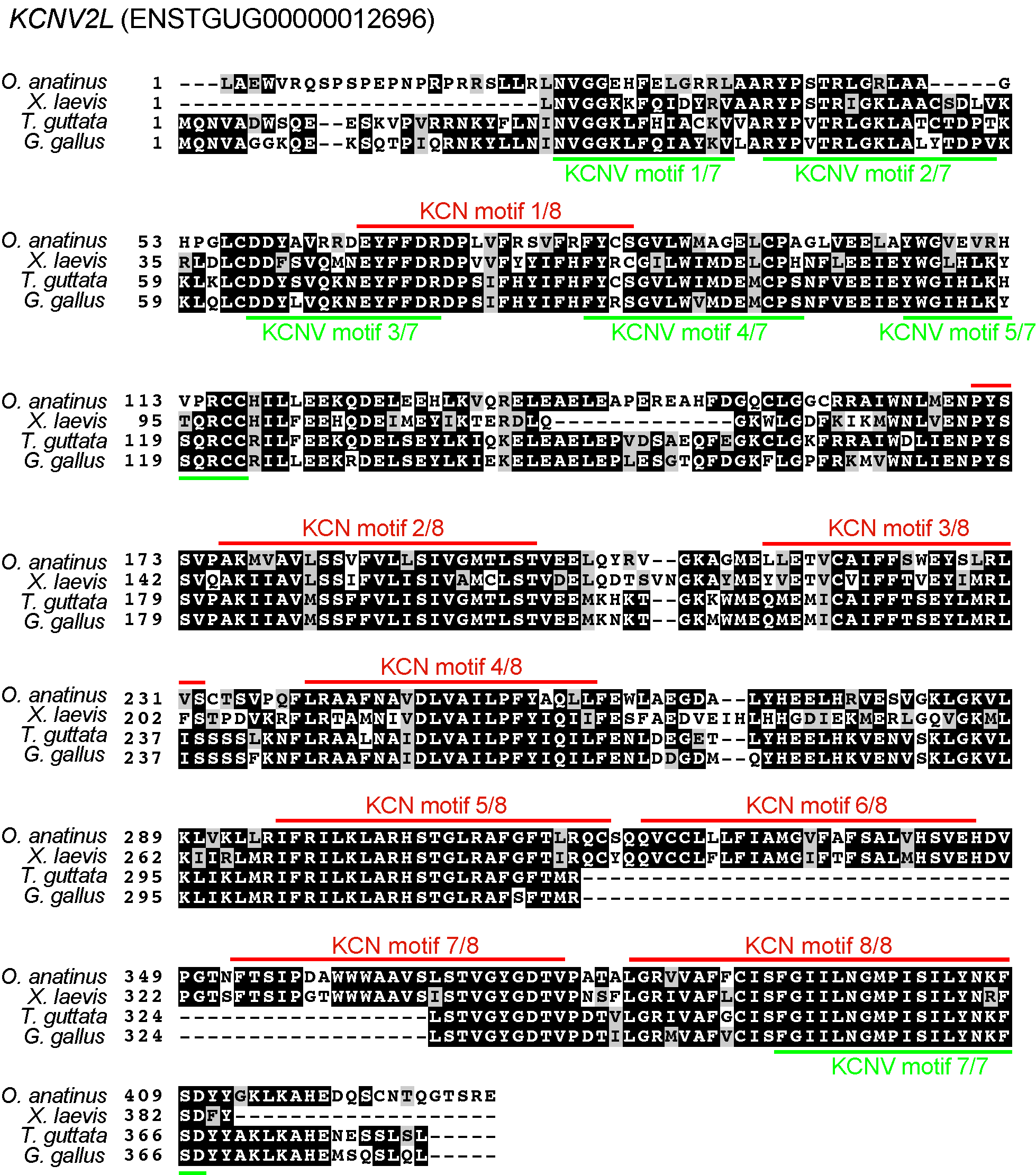

Supplement: Additional file 5 — Alignment of amino acid (AA) sequences predicted from KCNV2L orthologs in zebra finch, chicken, and frog. The numbers on the left indicate the relative position of AA residues in each sequence; AA residues are shaded in black when they are identical in >50% of species, and grey when the substitution is conservative. The positions of conserved KCN (voltage-gated) family sequence motifs are indicated in red; conserved sequence motifs that define the KCNV sub-family of channels are indicated in green. [file 1471-2164-14-470-S5.tiff]

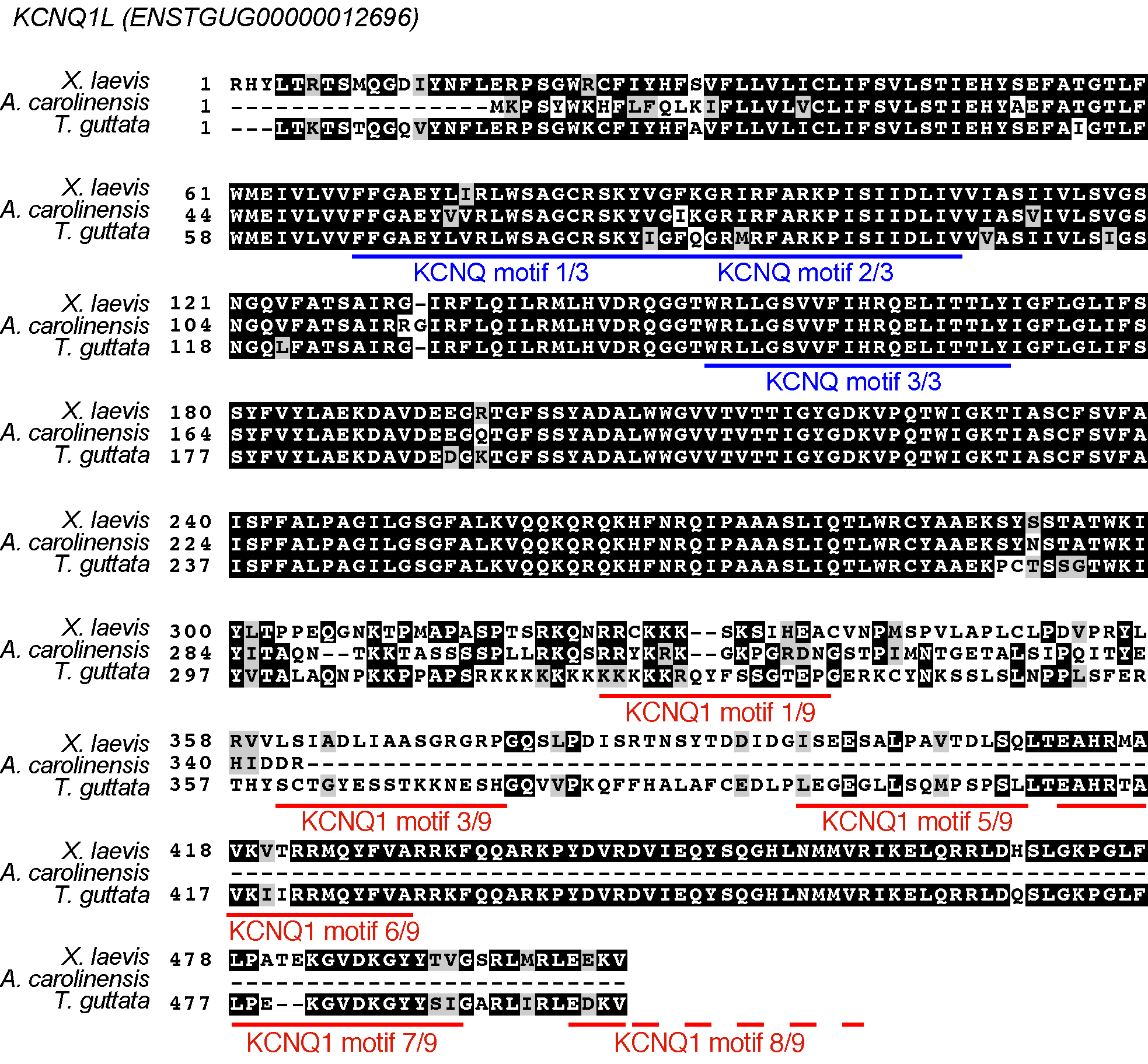

Supplement: Additional file 6 — Alignment of amino acid (AA) sequences predicted from KCNQ1L orthologs in zebra finch, chicken, and frog. The numbers on the left indicate the relative position of AA residues in each sequence; AA residues are shaded in black when they are identical in > 50% of species, and grey when the substitution is conservative. The positions of several of the nine conserved KCNQ Channel sequence motifs are indicated in red. [file 1471-2164-14-470-S6.tiff]
